# High expression level of ROR1 and ROR1-signaling associates with venetoclax resistance in chronic lymphocytic leukemia

**DOI:** 10.1038/s41375-022-01543-y

**Published:** 2022-04-13

**Authors:** Emanuela M. Ghia, Laura Z. Rassenti, Michael Y. Choi, Miguel Quijada-Álamo, Elvin Chu, George F. Widhopf, Thomas J. Kipps

**Affiliations:** 1 Moores Cancer Center, University of California, San Diego, La Jolla, CA 92093 USA; 2 Center for Novel Therapeutics, University of California, San Diego, La Jolla, CA 92037 USA; 3 Division of Hematology Oncology, Department of Medicine, University of California, San Diego, La Jolla, CA 92093 USA; 4 CIRM Alpha Stem Cell Clinic at University of California, San Diego and Sanford Stem Cell Clinical Center, La Jolla, CA 92037 USA; 5 University of Salamanca, IBSAL, IBMCC-CSIC, Cancer Research Center, Salamanca, Spain; 6 Department of Hematology, University Hospital of Salamanca, Salamanca, Spain

**Keywords:** Chronic lymphocytic leukaemia, Cancer therapeutic resistance

## Abstract

Although the BH3-mimetic venetoclax is highly cytotoxic for chronic lymphocytic leukemia (CLL) cells, some patients with CLL fail to clear minimal residual disease (MRD). We examined the CLL cells of seven such patients (CLL1-7) and found each had high-level expression of ROR1. By examining the CLL cells from such patients prior to therapy at SC1 and then more than 1 year later (Sample Collection 2 (SC2)), when they had progressive increases in MRD despite continued venetoclax therapy, we found the levels of ROR1 expressed on CLL cells at SC2 were significantly higher than that on CLL cells collected at SC1. At SC2, we also observed upregulation of genes induced by Wnt5a-induced ROR1 signaling, including *BCL2L1*. Transduction of the CLL-cell-line MEC1 to express *ROR1* enhanced expression of target genes induced by ROR1-signaling, increased expression of BCL-XL, and enhanced resistance to venetoclax, even in MEC1 made to express mutant forms of *BCL2*, which are associated with venetoclax resistance. Treatment of primary CLL cells with Wnt5a also increased their resistance to venetoclax, an effect that could be inhibited by the anti-ROR1 mAb (UC-961, zilovertamab). Collectively, these studies indicate that Wnt5a-induced ROR1-signaling can enhance resistance to venetoclax therapy.

## Introduction

Venetoclax is the first-in-class inhibitor of BCL2 that has been approved as treatment for patients with chronic lymphocytic leukemia (CLL) [1]. Treatment with venetoclax can affect deep clinical responses, often eradicating detectable minimal residual disease (MRD), particularly when used in combination with an anti-CD20 mAb [2–4], and/or inhibitors of BTK [5, 6]. Moreover, over two-thirds of treated patients lack detectable MRD in the blood or marrow after 1 year of venetoclax therapy. On the other hand, about a quarter of all patients fail to clear detectable MRD and are at risk for developing progressive disease (PD), even with continued therapy [5, 7–13].

Contributing to drug resistance is the acquisition of mutations in BCL2 affecting its capacity to bind venetoclax [14–16]. The CLL cells from each of several venetoclax-resistant patients have mutations in *BCL2*, most commonly *BCL2*^G101V^, generated by a nonsynonymous mutation affecting a glycine to valine substitution at amino-acid-position 101 of *BCL2*. Venetoclax binds poorly to the mutant BCL2 encoded by *BCL2*^G101V^ and thus is less able to induce CLL-cell apoptosis. For unexplained reasons, however, the *BCL2*^G101V^ mutation alone is rarely found at allelic frequencies greater than 25% in the leukemia cells of patients who are resistant to venetoclax. For example, Blombery and colleagues described 11 patients with progressive CLL who had leukemia cells with *BCL2*^G101V^ and other oligoclonal *BCL2* mutations [17]. However, only four of these patients had proportions of CLL cells harboring *BCL2* mutations that appeared greater than 50%. As such, factors other than BCL2 mutations also apparently contribute to venetoclax resistance.

With that in mind, recent studies found that CLL cells of refractory patients appeared more resistant to the cytotoxic effects of venetoclax when cultured with accessory cells found in the leukemia microenvironment [15, 18]. Factors provided by the leukemia microenvironment, such as Wnt5a [19], may enhance resistance to venetoclax. Relevant in this regard is the leukemia-cell expression of the receptor tyrosine kinase-like orphan receptor (ROR1), which can serve as a receptor for Wnt5a [20]. Wnt5a can induce ROR1-signaling leading to expression of ERK1/2 and NF-κB target genes [19, 21], the expression of which may mitigate the cytotoxicity of venetoclax [22]. Moreover, expression of ROR1 in mouse models of CLL enhances activation of signaling networks that induce expression of genes implicated in embryonic- and tumor-cell proliferation and survival [23].

The CLL cells from over 90% of all patients express ROR1; [20, 24] however, there is heterogeneity in the expression levels of ROR1 on leukemia cells among different patients [24]. The CLL cells that express high levels of ROR1 (*ROR1*^*Hi*^ CLL) have differences in gene expression from CLL cells that express low-to-negligible levels of ROR1 (*ROR1*^*Lo*^ CLL); such gene expression differences also are observed between ROR1-expressing leukemia cells that develop in Eµ-ROR1/TCL1-trangenic mice versus the ROR1-negative leukemia cells of Eµ-TCL1 transgenic mice [20, 24]. More recently, genes targeted through activation of ROR1 signaling (e.g., ERK1/2 or NF-κB target genes) have been defined [19, 21], including the *BCL2L1* gene encoding BCL-XL, which may enhance resistance to venetoclax in CLL or mantle-cell-lymphoma [25–28]. Consistent with this notion, Blombery and colleagues found a case of venetoclax-resistant CLL that lacked detectable *BCL2* mutations, but had high levels of BCL-XL [15].

We hypothesize that ROR1-signaling may enhance expression of genes that enhance resistance to cytotoxic drugs; a corollary of this hypothesis is that inhibition of ROR1-signaling may mitigate resistance to therapy. In this study, we examined the leukemia-cell expression of ROR1 and ROR1-signaling of patients who failed to clear MRD after more than a year of therapy with venetoclax.

## Materials and methods

### Patients samples

Samples from patients who satisfied diagnostic criteria for CLL [29], were collected in accordance with the Declaration of Helsinki for the protection of human subjects and the Institutional Review Board (IRB) of the University of California San Diego (IRB approval number 110658).

### Immunophenotyping

ROR1 expression levels were assessed via flow cytometry, as described [30]. The expression of ROR1 was detected using Alexa-647-conjugated anti-ROR1 mAb (4A5) (BD Biosciences). CLL cells also were analyzed for CD19, CD20, and CD23, using mAbs conjugated to allophycocyanin (APC), Peridinin-chlorophyll-protein (PerCp), fluorescein isothiocyanate (FITC), or phycoerythrin (PE), respectively (BD Biosciences). Fluorochrome-conjugated, isotype control mAbs of irrelevant specificity were used to monitor for nonspecific staining. The relative expression levels of ROR1 were reported as absolute mean fluorescence intensity (AbMFI), which is determined by subtracting the mean fluorescence intensity of the CLL cells stained with a nonspecific antibody of the same isotype from the mean fluorescence intensity of the CLL cells stained with the anti-ROR1 antibody. Data were acquired on a FACSCelesta (BD Biosciences) and analyzed using FlowJo software (v.9.3.2. FlowJo).

### RNA extraction and RNA-Seq

Negative isolation of CLL cells to ≥95% purity was performed prior to extraction of RNA using RNeasy Plus Micro Kit (Qiagen). We performed RNA sequencing with sequencing depth of at least 30 million reads per sample to assure 90–95% sensitivity in detecting variants [31]. Data were analyzed by Rosalind (https://rosalind.onramp.bio/), with a HyperScale architecture developed by OnRamp Bioinformatics, Inc. (San Diego, CA). Reads were trimmed using cutadapt [32]. Quality scores were assessed using FastQC [33]. Reads were aligned to the Homo sapiens genome build hg19 using STAR [34]. Individual sample reads were quantified using Htseq [35] and normalized via Relative Log Expression (RLE) using DESeq2 R library [36]. DEseq2 was also used to calculate fold changes and p-values. The accession number for the RNA-seq data reported in this paper is GSE192685 (http://www.ncbi.nlm.nih.gov/geo/).

### Gene-set-enrichment analysis

We performed gene-set-enrichment analysis (GSEA) on ERK1/2, and NF-κB target genes and on genes induced by binding of a Wnt protein to a frizzled family receptor on the surface of the target cell, followed by propagation of the signal via beta-catenin (canonical Wnt signaling pathway) or via effectors other than beta-catenin (non-canonical Wnt signaling pathway) [19, 21, 37–42]. Each gene set was considered significant when the false discovery rate (FDR) was less than 25% [43]. The FDR q value was adjusted for gene set size and multiple hypothesis testing.

### Immunoblot analysis

Cells were lysed using RIPA lysis buffer. Protein concentration was determined using the DC (Detergent compatible) protein assay (BioRad, Hercules, CA, USA). Equal amounts of protein lysates (20 μg) were separated by gel electrophoresis using a NuPAGE Novex 4–12% Bis-Tris Midi Gel (Invitrogen) and transferred to nitrocellulose or polyvinylidene fluoride membranes. Immunoblots were probed using anti-BCL-XL antibody (Cat#2764), anti-BCL2 antibody (Cat#4223) from Cell Signaling Technology (Danvers, MA), or anti-β-actin (sc-47778) from Cell Santa Cruz Biotechnology (Dallas, TX).

### In vitro viability assay

MEC1 and MEC1-ROR1 viability in response to venetoclax treatment was assessed using CellTiter-Glo 2.0 Assay (Promega). Cells were seeded in black 96-well plates at a concentration of 1 × 10^5^ cells/well and treated with escalating doses of venetoclax. Viability was measured 24 h after treatment using a Tecan Infinite M200 plate reader (Tecan).

### Ex vivo viability experiments

ROR1-high primary CLL cells were cultured in RPMI media (Life Technologies) supplemented with 10% FBS at a density of 4 × 10^6^ cells/mL. CLL cells were cultured in serum-free media, with or without UC-961 (zilovertamab at 20 μg/mL) and with or without exogenous Wnt5a (200 ng/mL, R&D Systems). Venetoclax was added at concentrations ranging from 0.5 nM to 4 nM. Viability at 16 h was determined as the percentage of viable cells relative to that observed in cultures treated with DMSO without venetoclax, as described [30].

### *BCL2* constructs and transfection assays

Green-fluorescent protein (GFP)-tagged pRP mammalian gene expression vectors encoding either *BCL2*^WT^, *BCL2*^A113P^, or *BCL2*^G101V^ (VectorBuilder, Shenandoah, TX) were transfected into MEC1 or MEC1-ROR1 cell lines using Neon Transfection System (ThermoFisher Scientific). For this, 2 × 10^6^ cells were washed in PBS and re-suspended in 100 μL of R solution. 12.5 μg of each plasmid were transfected using the following parameters: 1350 V; 30 ms, 1 pulse. GFP expression was assessed at 24 h by flow cytometry as a control to monitor the efficiency of transfection. At that time, transfected cells were treated with DMSO or venetoclax (5 µM). After 24 h, the cells were stained with Annexin V-PE (BioVision) for 5 min and then analyzed by flow cytometry. For each plasmid, we determined the percentage of specific venetoclax-induced apoptosis of the transfected cells (% GFP^+^ Annexin^+^).

### Statistical analyses

Statistical analysis was carried out using GraphPad Prism software v6 (GraphPad Software) and *p*-values were determined using the paired Student’s *t* test and considered significant with a *p*-value of less than 0.05.

## Results

### Assessment Of ROR1 And ROR1-regulated target genes before and after development of resistance to venetoclax

**Fig. 1:**
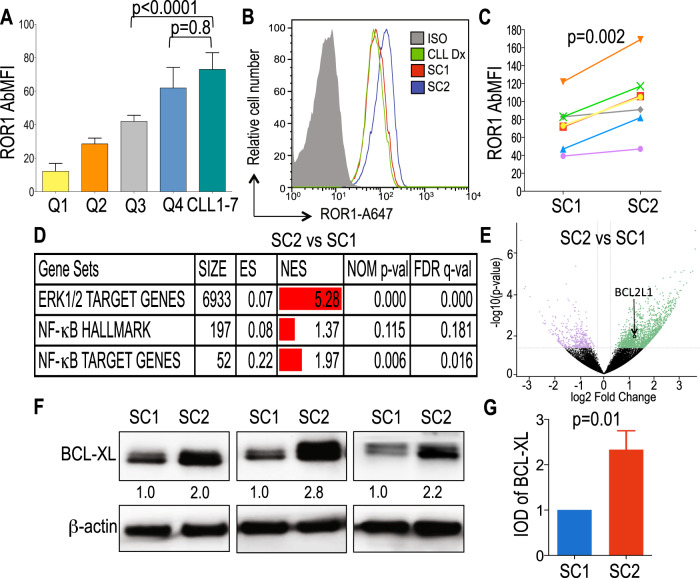
Expression of ROR1 and ROR1-regulated target genes. **A** Data are shown as the median with interquartile range of ROR1 expression on CLL cells in a cohort of 1,568 cases [24] divided in 4 quartiles (Q1, Q2, Q3, and Q4) each comprised 392 CLL cases and in patients CLL1-7. **B** Representative histograms depict the fluorescence of cells stained with an isotype control mAb (gray) or anti-ROR1 (4A5) at diagnosis (green, CLL Dx), at SC1 (red), and at SC2 (blue). **C** ROR1 expression reported as AbMFI measured by flow cytometry prior to therapy (SC1) and at MRD progression on venetoclax therapy (SC2). **D** GSEA of the genes expressed in negatively-selected CLL cells collected at MRD progression on venetoclax therapy (SC2) versus those expressed by the negatively-selected CLL cells collected from the same patients prior to treatment (SC1). GSEA on the transcriptomes of CLL cells collected at SC2 versus SC1, evaluating for differences in the expression of a set of genes associated with ROR1 regulated pathways [19, 21, 39]. Gene-set size (SIZE), enrichment score (ES), normalized ES (NES), nominal *p* value (NOM p-val), and FDR *q* value (FDR q) are indicated. **E** Volcano plot showing differences in gene expression between SC2 versus SC1. The log2 of the fold change (log2 Fold Change) is on the *X* axis, and the negative log10 of *p*-value (-log10 *p*-Value) is on the *Y* axis. Vertical dashed lines indicate fold change of 1.2 and −1.2, respectively. Horizontal dashed line indicates a *p*-value of 0.05. Each dot represents a gene within the comparison performed. The coloring on the dots reflects whether each gene is significantly overexpressed (green) or under-expressed (purple) in SC2 versus SC1, and those in black are genes that were not significantly overexpressed or under-expressed in SC2 versus SC1. The significant overexpression of *BCL2L1* at SC2 is indicated. **F** BCL-XL protein expression levels assessed by immunoblot analysis at SC1 and SC2 in three cases. The membranes were probed with a monoclonal antibody specific for BCL-XL or β-actin, as indicated on the left margin. The density of the β-actin band was used to normalize band density for BCL-XL for each sample. The integrated optical density (IOD) ratios of the band densities of BCL-XL/β-actin normalized with respect to SC1 for each sample are indicated at the bottom of BCL-XL immunoblots and presented in the bar graph in panel H. **G** The IOD ratios of BCL-XL are shown as the mean ± SD of SC1 or SC2 of three samples. Statistical significance was determined by Paired Student’s *t* test.

We studied the CLL cells of seven patients who were treated with venetoclax-based therapy, but failed to clear detectable minimal residual disease (MRD) and then developed progressive increases in MRD while continuing therapy with venetoclax, after a median of 2 years of treatment (Supplementary Fig. S1). Prior to venetoclax therapy, these patients had relapsed disease with a median of three prior therapies (range 1–5). The CLL cells of six patients used unmutated immunoglobulin heavy chain genes (U-IGHV) and the CLL cells of the 7th patient used a lightly mutated IGHV (M-IGHV) encoded by IGHV3-21 (Table 1). We assessed for expression of ROR1 by flow cytometry on CLL cells obtained from patients CLL1-7 prior to therapy at (Sample Collection 1 (SC1)). We found each expressed high levels of ROR1, with a mean level comparable to that of CLL cells within the top 25th percentile of ROR1 among 1568 patients examined in our reported cohort (top quartile, *n* = 392) (Fig. 1A) [24]. The probability that all seven samples would express ROR1 in the top quartile is (0.25)^7^, or 0.00006.

**Table 1:** Clinical and biological characteristics of CLL samples collected from seven patients (CLL1-7) prior to venetoclax therapy.

Sample ID	Gender	IGHV status (%)	IGHV gene	FISH (%)	Karyotype	List of all therapies prior venetoclax	Age at CLL DX	SC2 time point from Venetoclax start date (yrs)	% increase in MRD at SC2 from lowest detectable MRD achieved	Time on venetoclax therapy (yrs)	Type of venetoclax therapy
CLL1	Male	U-IGHV (100%)	IGHV3-48	normal	Abnormal: 46, XY, t(1;14)(q42;q32)[cp8]/46,XY[12]	(1) FCR, (2) Campath, (3) HDMP, (4) Campath	48.3	5.2	50	6.4	Venetoclax alone
CLL2	Female	U-IGHV (100%)	IGHV1-69	tri 12 (53%)	Abnormal: 47, XX, +12[13]/47, idem, del(8)(p11.2)[3]	(1) FR, (2) Revlimid+Rituximab, (3) Revlimid Consolidation, (4) Ofatumumab, (5) Ibrutinib	63.1	1.1	30	1.2	Venetoclax alone
CLL3	Male	U-IGHV (99.7%)	IGHV1-69	13q del (11.5%), 17p del (11.5%)	Complex: 46, XY, del(13)(q12q14)[2]/43, X, −Y, add(1)(q42), add(4)(?q25q31), del(12)(p13), −13, der(17)t(17;18)(p11.2; q11.2), −18[1]/46,XY[17]	(1) Ad-ISF35 Intranodal Injection + FCR	55.4	4.9	49	6.5	Venetoclax + anti-CD20
CLL4	Male	U-IGHV (100%)	IGHV1-2	13q del (64%), 17p del (70%)	Complex: 46, XY, del(13)(q13q14)[1]/46, idem, add(2)(p13), del(7)(q?34q36), −8, der(15)t(8;15)(q?21; p11.2), del(17)(p12), +mar[cp7]/46, idem,der(7)t(7; 12)(q22; q24.1), der(12)add(12)(p12)t(7; 12)(q22; q24.1)[cp7]/46, XY[7]	(1) FC, (2) HDMP, (3) Ibrutinib	67.6	0.7	1	0.7	Venetoclax alone
CLL5	Female	U-IGHV (100%)	IGHV3-33	13q del (10%), 17p del (8%)	Complex: 45, XX, del(1)(q42), add(4)(p15), del(6)(q21), −8, add(10)(p?13), −13, del(17)(p13), add(18)(q12), +mar[cp8]/46,XX[14]	(1) FCR, (2) AVL-292, (3) Ibrutinib	52.6	1.8	83	1.8	Venetoclax alone
CLL6	Male	U-IGHV (99.7%)	IGHV1-69	11q del (56%), 17p del (59%)	Complex: 46, XY, t(8; 9)(q24;q22), ?11q[3]/45, der(8)t(8;9)(q24; q22), −9, ?11q, der(17)t(9; 17)(p?13;p11.2)[10]/46,XY[9]	(1) Revlimid+Rituximab, (2) HDMP+Ofatumumab, (3) Revlimid+Rituximab, (4) Ublituximab + Ibrutinib, (5) Ibrutinib	56.3	1.4	40	1.5	Venetoclax alone
CLL7	Male	M-IGHV (97.9%)	IGHV3-21	13q del (23%), 11q del (6%), 17p del (16%)	Complex: 45, XY, del(9)(p?21), der(9)t(9; 21)(?q11;?q11), del(17)(p11.2), −21[3]/44, idem, −5, add(14)(p11.2)[4]/46,XY,del(11) (?q22q25)[4]/nonclonal abnormality[1]/46, XY[14].nuc ish(ATMx1)[11/200], (CCND1,IGH)x2[200], (D12Z3x2) 200], (D13S319x0)[46/200], (LAMP1x2)[200], (TP53x1)[31/200]	(1) FCO, (2) Navitoclax + BR, (3) Obinutuzumab	58.5	2.8	49	3.8	Venetoclax alone

Percent (%) increase in MRD at SC2 from lowest detectable MRD achieved calculated with the formula 100 x [(Log10(%MRD x ALC at SC2) - [(Log10(%MRD x ALC at 12 months)/[(Log10(%MRD x ALC at 12 months). *FCR* Fludarabine+Cyclophosphamide+Rituximab, *FR* Fludarabine+Rituximab, *HDMP* High Dose Methylprednisolone, AVL-292: Btk inhibitor, *FC* Fludarabine+Cyclophosphamide, *BR* Bendamustine+Rituximab, *FCO* Fludarabine+Cyclophosphamide+Ofatumumab.

We compared the expression levels of ROR1 on CLL cells from these patients before therapy at SC1 with that of CLL cells obtained more than 1 year later (Sample Collection 2 (SC2)), when they were noted to have progressive increases in MRD despite continued venetoclax therapy (Fig. 1B). The mean level of ROR1 expressed by CLL cells at SC2 was significantly higher than that of the CLL cells from the same patients collected at SC1 (*p* = 0.002, Paired *t* test) (Fig. 1C).

### Assessment of ROR1-regulated target genes in ROR1^Hi^ and ROR1^Lo^ CLL

We examined the transcriptomes of CLL cells from patients with ROR1^Lo^ CLL cells (*N* = 12) versus those with ROR1^Hi^ CLL (*N* = 12) for their relative expression of ERK1/2 and NF-κB target genes [19, 21, 39]. This analysis revealed enrichment of ERK1/2 and NF-κB target genes, including *BCL2L1*, in ROR1^Hi^ cases relative to that of ROR1^Lo^ cases (Supplementary Fig. S2).

We also examined the transcriptomes of negatively-selected CLL cells from patients who developed venetoclax resistance at SC1 and SC2. The GSEA of the transcriptomes of CLL cells for each patient also demonstrated significant increases at SC2 relative to SC1 in the expression of genes targeted by activation of ERK1/2, NF-κB, and non-canonical Wnt signaling [19, 21, 37–42] (Fig. 1D, Supplementary Table S1). However, we did not observe a significant increase at SC2 relative to SC1 in the expression of genes induced by activation of the canonical β-catenin/Wnt-signaling pathway (Supplementary Table S1). Moreover, for each patient, we found that the CLL cells at SC2 had significantly higher levels of *BCL2L1* and BCL-XL protein than the CLL cells at SC1 (*p* = 0.009 and *p* = 0.01, respectively) (Fig. 1E–G, Supplementary Table S2).

### Venetoclax resistance in MEC1 and MEC1-ROR1

**Fig. 2:**
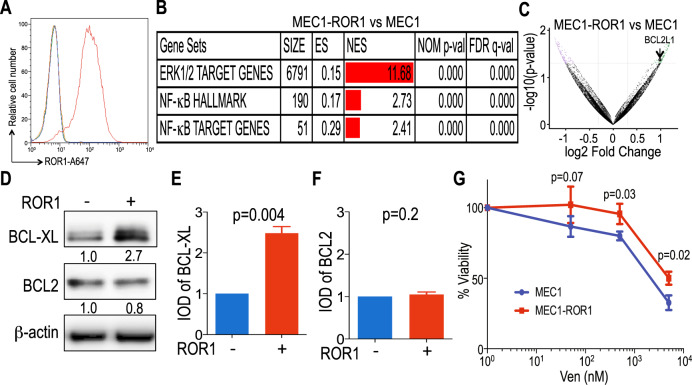
Relative expression of ROR1-regulated target genes and venetoclax sensitivity of MEC1 versus MEC1-ROR1. **A** ROR1 protein expression levels (AbMFI) measured by flow cytometry on MEC1 and MEC1-ROR1 cells labeled with A647-conjugated anti-ROR1 mAb (blue and red histograms, respectively) or A647-conjugated nonspecific IgG of the same isotype (green and orange histograms). **B** GSEA on the transcriptomes of MEC1 versus MEC1-ROR1, evaluating for differences in the expression of genes associated with ROR1 regulated pathways [19, 21, 39]. Gene-set size (SIZE), enrichment score (ES), normalized ES (NES), nominal *p* value (NOM p-val), and FDR *q* value (FDR q) are indicated. **C** Volcano plot showing differences in gene expression between MEC1-ROR1 versus MEC1. The log2 of the fold change (Log2 Fold Change) is on the *X* axis, and the negative log of *p*-value (-Log10 *p*-Value) is on the *Y* axis. Vertical dashed lines indicate fold change of 1.2 and −1.2, respectively. Horizontal dashed line indicates a *p*-Value of 0.05. Each dot represents a gene within the comparison performed. The coloring on the dots reflects whether each gene is significantly overexpressed (green) or under-expressed (purple) in MEC1-ROR1 versus MEC1, and those in black are genes that were not significantly overexpressed or under-expressed in MEC1-ROR1 versus MEC1. The significant overexpression of *BCL2L1* gene in MEC1-ROR1 is indicated. **D** BCL-XL and BCL2 protein expression levels assessed by immunoblot analysis on MEC1 and MEC1-ROR1 cells. The membranes were probed with a monoclonal antibody specific for BCL-XL, BCL2, or β-actin, as indicated on the left margin. The density of the β-actin band was used to normalize band density for BCL-XL or BCL2. The IOD ratios of the band densities of BCL-XL/β-actin normalized with respect to that of MEC1 cells are indicated at the bottom of BCL-XL immunoblots and presented in the bar graph in panel (**E**). The IOD ratios of the band densities of BCL2/β-actin normalized with respect to that of MEC1 cells are indicated at the bottom of BCL2 immunoblots and presented in the bar graph in panel (**F**). **E**, **F** The IOD ratios of BCL-XL (**E**) or BCL2 (**F**) are shown as the mean ± SD of three independent immunoblots. Statistical significance was determined by Student’s *t* test. **G** MEC1 (blue) and MEC1-ROR1 (red) cells were treated with different concentrations of venetoclax (0.05 μM to 5 μM) and cell viability was assessed by CellTiter-Glo viability assay at 24 h. The fraction surviving venetoclax therapy is expressed relative to that of the untreated (DMSO) control. Data are summarized as the mean ± SEM of three independent experiments.

Prior studies found that the CLL-derived cell line MEC1 lacks expression of ROR1 [44], but has constitutive expression of Wnt5a, which can induce ROR1-signaling [20]. MEC1 cells were transduced to express ROR1 to generate MEC1-ROR1 cells (Fig. 2A). Transcriptome analyses revealed that MEC-ROR1 had enhanced expression of ERK1/2 and NF-κB target genes [19, 21, 39], including *BCL2L1*, relative to that of MEC1 cells (Fig. 2B, C, Supplementary Table S3). Moreover, we observed a significant increase in MEC1-ROR1 relative to MEC1 cells in their expression of genes induced by activation of non-canonical Wnt signaling pathways, but not genes induced through activation of the canonical β-catenin/Wnt signaling pathway [38] (Supplementary Table S3). Relative to MEC1 cells transduced with a control vector, MEC1-ROR1 expressed significantly higher levels of BCL-XL, but comparable levels of BCL2 (*p* = 0.004 and *p* = 0.2, respectively) (Fig. 2D–F). Collectively, these results indicate that expression of ROR1 was sufficient to enhance expression of NF-κB target genes, including *BCL2L1*, in MEC1 leukemia cells.

MEC1 leukemia cells already are relatively resistant to venetoclax compared to the primary leukemia cells of patients with CLL [45]. Nonetheless, we examined whether the expression of ROR1 could enhance the resistance of this cell line to venetoclax in vitro. For this we cultured MEC1 and MEC-ROR1 in media containing venetoclax at concentrations ranging from 0.05 μM to 5 μM. We found that MEC1-ROR1 cells had a significantly higher viability than MEC1 cells at each concentration of venetoclax examined (Fig. 2G).

### Evaluation of *BCL2* mutations before or after development of venetoclax resistance

We also examined for *BCL2* mutations in the CLL cells collected at SC1 and SC2 from each of the seven patients who developed resistance to this drug. We identified the *BCL2*^*G101V*^ mutation in the CLL cells collected at SC2 from each of three patients at allelic frequencies of less than 20%. Moreover, one of these three patients had subclonal co-occurrence of this *BCL2*^*G101V*^ mutation together with a *BCL2*^*A113G*^ mutation, affecting an amino acid change of A-to-G at position 113 of *BCL2*. Each of these *BCL2* mutations were found at allelic frequencies of less than 25% (Supplementary Table S4) [17]. These mutations were not detected in any of the patient samples collected at SC1.

**Fig. 3:**
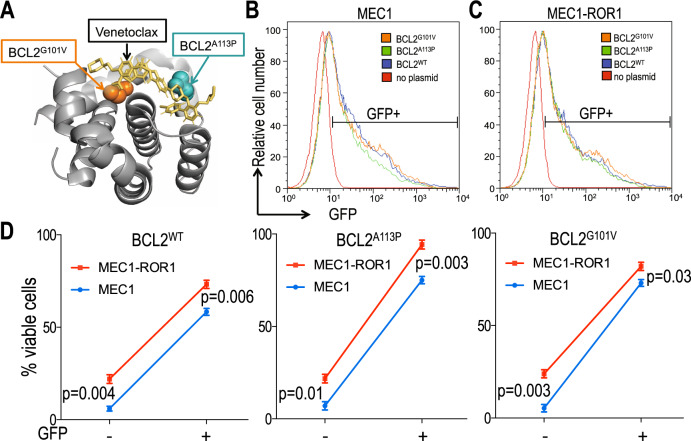
Relative sensitivity of MEC1 versus MEC1-ROR1 cells following transfection with GFP/BCL2-expression vectors. **A** Ribbon representation of α-helices that form the BCL2 binding groove, indicating the locations of Gly101 (orange spheres) and A113 (teal spheres). The structure is that of venetoclax analogue (yellow) bound to BCL2 (PDB:4MAN). **B**, **C** Representative histograms show the GFP fluorescence of MEC1 (**B**) or MEC1-ROR1 (**C**) cells transfected with BCL2 variants (blue, green, and orange histograms, respectively) or control (red histograms). **D** Average percent of viable GFP- or GFP+ cells in MEC1 or MEC1-ROR1 following treatment with 5 μM venetoclax. MEC1 (blue) or MEC1-ROR1 (red) were transfected with BCL2^WT^, BCL2^A113P^, or BCL2^G101V^. The percent of viable cells after 24 h treatment is indicated. Data are shown as the mean ± SEM of five independent experiments. Statistical significance was determined by Student’s *t* test.

In the CLL cells of a fourth patient, we found a non-reported nonsynonymous *BCL2* mutation (*BCL2*^*A113P*^) that results in an alanine to proline substitution at amino acid position 113 of *BCL2*. Review of the known tertiary structure of BCL2 revealed that this mutation causes a substitution of proline for an alanine at a position contiguous to the BH3-binding pocket (Fig. 3A). Unlike the other *BCL2* mutations identified in the three aforementioned samples, *BCL2*^*A113P*^ was detected at an allelic frequency of 49.3%, indicating that virtually all of the leukemia cells within this sample harbored this mutation. This mutation, however, was not identified in the CLL cells collected from this patient at SC1 (Supplementary Table S4). Finally, in three of the seven CLL cases, we did not identify any mutations in *BCL2* at SC2, as noted for the CLL cells of some patients resistant to venetoclax [17].

We transfected MEC1 versus MEC1-ROR1 cells with GFP-tagged plasmids encoding either wild type (WT) *BCL2 (BCL2*^*WT*^*)* or one of the mutant *BCL2* identified in the CLL cells of patients resistant to venetoclax and assessed the capacity of each to enhance the resistance of these cells to treatment with venetoclax in vitro. We assessed the cells for expression of GFP at 24 h after transfection by flow cytometry; this revealed the transfected MEC1 or MEC1-ROR1 cells each expressed highly comparable amounts of GFP encoded by each of these three different plasmids, which encoded *BCL2*^*WT*^, *BCL2*^*A113P*^, or *BCL2*^*G101V*^ (Fig. 3B, C). Transfection with each of the three different plasmids encoding *BCL2*^*WT*^, *BCL2*^*A113P*^, or *BCL2*^*G101V*^ did not change the BCL-XL protein expression ratios in MEC1-ROR1 cells relative to that in MEC1 cells (Supplementary Fig. S3). We examined the sensitivity of each of these transfected cells to the cytotoxic effects of venetoclax added to a final concentration of 5 µM in the culture media in each of five separate experiments. We observed GFP^+^ MEC1 cells or MEC1-ROR1 cells transfected with *BCL2*^*WT*^, *BCL2*^*A113P*^ or *BCL2*^*G101V*^ had significantly higher viability following treatment with venetoclax than GFP-negative MEC1 cells or MEC1-ROR1 cells that lacked expression of the plasmid transgenes (e.g., *BCL2*^*WT*^, *BCL2*^A113P^, or *BCL2*^*G101V*^) (Fig. 3D). However, MEC1-ROR1 cells had higher viability than MEC1 cells independent of whether they expressed the plasmid transgenes. Collectively, these results indicate that expression of ROR1 can enhance the viability of the venetoclax-treated leukemia cells, including leukemia cells that expressed mutant forms of *BCL2* associated with venetoclax resistance.

### Effect of zilovertamab on Wnt5a-induced venetoclax resistance of ROR1^Hi^ CLL cells

**Fig. 4:**
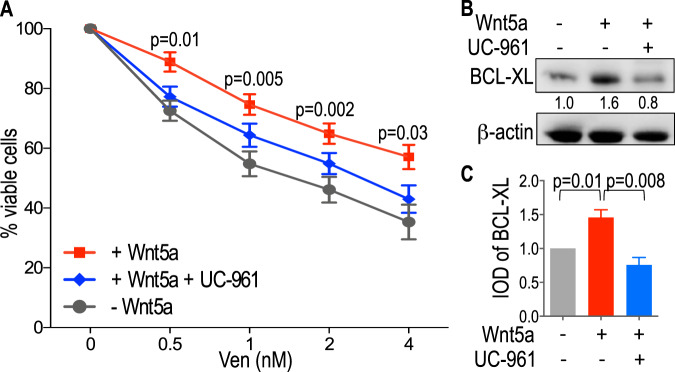
Zilovertamab inhibits Wnt5a-induced venetoclax-resistance and expression of ROR1-regulated target genes in CLL cells. **A** CLL cells expressing ROR1 (*n* = 13) were cultured in serum-free media and treated with increasing venetoclax doses (0.5 nM to 4 nM), with or without zilovertamab (UC-961, 20 μg/mL) in the presence or absence of exogenous Wnt5a (200 ng/mL). CLL cell viability is represented as the percentage of cells after 16 h of treatment with venetoclax or carrier (DMSO) for each sample. Data are shown as mean ± SEM of three independent experiments. For each dose of venetoclax (Ven), statistical significance was determined by two-way Anova, by comparing the percentage of viable cells after 16 h of treatment with Wnt5a (+Wnt5a) minus the percentage of viable cells after 16 h with Wnt5a and zilovertamab (+Wnt5a +UC-961). **B** BCL-XL protein expression levels assessed by immunoblot analysis of lysates prepared from ROR1^Hi^ CLL cells (representative of three patients) treated without (−) or with (+) Wnt5a and without (−) or with (+) zilovertamab (UC-961). The membranes were probed with a monoclonal antibody specific for BCL-XL or β-actin, as indicated on the left margin. The density of the β-actin band was used to normalize band density for BCL-XL. The IOD ratios of the band densities of BCL-XL/β-actin normalized with respect to that of CLL cells without Wnt5a or zilovertamab (UC-961) are indicated at the bottom of BCL-XL immunoblots and presented in panel G. **C** The IOD ratios of BCL-XL are shown as the mean ± SD of three samples without (−) or with (+) Wnt5a and without (−) or with (+) zilovertamab (UC-961). Statistical significance was determined by Paired Student’s *t* test.

We examined whether treatment of CLL cells with Wnt5a, with or without the anti-ROR1 mAb zilovertamab, could affect their sensitivity to venetoclax. For this, we cultured CLL cells overnight in serum-free media with carrier protein, with or without recombinant Wnt5a, and with or without zilovertamab. Treatment with Wnt5a enhance the resistance of CLL cells to venetoclax at all concentrations tested (Fig. 4A), an effect that could be inhibited by zilovertamab (Fig. 4A). Immunoblot analysis of lysates of CLL cells treated without or with recombinant Wnt5a and without or with zilovertamab revealed that Wnt5a treatment increased BCL-XL protein expression and that zilovertamab inhibited Wnt5a-induced increases in BCL-XL (Fig. 4B, C). Collectively, these studies indicate that Wnt5a can enhance resistance of ROR1^Hi^ CLL cells to venetoclax by increasing BCL-XL expression, and that this effect could be inhibited by this anti-ROR1 mAb.

## Discussion

In this study, we found that the pre-treatment CLL cells of seven patients who subsequently failed to clear MRD each expressed levels of ROR1 comparable to those in the top 25th percentile of a large cohort of patients with CLL, indicating a significant association between the subsequent failure to clear MRD with high-level expression of ROR1 prior to therapy (*p* = (0.25)^7^, or 0.00006). Furthermore, we found the CLL cells collected ≥1 year of venetoclax therapy had even higher levels of ROR1 than did the pre-treatment CLL cells of each patient. These observations reveal an association between high-level leukemia-cell expression of ROR1 and failure to clear detectable MRD after a year or more of venetoclax therapy.

CLL cells that express high levels of ROR1 also are noted more commonly to express unmutated IGHV, have complex karyotypes, and/or inactivating mutations in *TP53* than CLL cells with low-ROR1 [24]. Such features also are associated with venetoclax resistance [46], raising the question of whether such features instead account for the noted association between high-level ROR1 expression and venetoclax resistance. However, it should be noted that patients with CLL cells with high-level expression of ROR1 have accelerated disease progression and shorter overall survival than patients with low-to-negligible levels of ROR1 independent of IGHV mutation status [24]. Similarly, there also may be an independent causal relationship between high-level expression of ROR1 and development of venetoclax resistance, leading us to hypothesize that ROR1-signaling per se may mitigate the cytotoxicity of venetoclax [47].

Our analysis of CLL cells before (SC1) and after 1 or more years of venetoclax therapy (SC2) showed the CLL cells at SC2 expressed even higher levels of ROR1 than the CLL cells collected at SC1. We found that such higher-level expression of ROR1 also associated increased expression of ERK1/2- and NF-κB- target genes, which our prior studies found could be induced by Wnt5a-ROR1 signaling [19, 21]. We identified *BCL2L1*, encoding the BCL-XL protein, *a*mong the NF-κB- target genes associated with expression of ROR1. The increased expression of *BCL2L1* induced by Wnt5a-activation of ROR1 may account in part for the enhanced resistance to venetoclax of leukemia cells that express high levels of ROR1. Furthermore, we found that CLL cells at SC2 had higher expression of genes implicated in the non-canonical Wnt signaling, but not genes induced through activation of the canonical β-catenin/Wnt signaling pathway; we found changes in gene expression similar to those in SC2 versus SC12 in MEC1-ROR1 versus MEC1 cells, indicating that ROR1 alone was sufficient to induce such changes in gene expression for this cell line, which has constitutive high-level expression of Wnt5a.

It should be emphasized that high-level expression of ROR1 is not solely responsible for venetoclax resistance. As noted in prior studies [15, 17, 48, 49], our study also revealed that *BCL2*^*G101V*^ mutations in the CLL cells collected at SC2 were present in three out of seven patients and that each of these mutations were found at allelic frequencies less than 25%. In addition to the Gly101Val codon variant, subclonal mutation Ala113Gly also was observed in the CLL cells of one patient, as noted in other patients with drug resistance [17, 50]. We also identified a previously non-reported nonsynonymous *BCL2* mutation (*BCL2*^*A113P*^) at an allelic frequency of 49.3%, which in contrast to previously noted mutations was present at an allelic frequency indicating that nearly all the CLL cells of this patient expressed this mutant allele of *BCL2*. Like the other previously identified mutations in *BCL2* associated with venetoclax resistance, this mutation encodes a residue close to the site bound by venetoclax, thereby potentially mitigating the capacity of this drug to bind and inhibit BCL2. Because the *BCL2*^*A113P*^ was found at an allelic frequency of 49.3%, this mutant form of *BCL2* was found in virtually all CLL cells at SC2, but in none of the CLL cells at SC1, demonstrating strong selection of this mutant *BCL2* in the treated CLL population. However, the wide range of allelic frequencies of *BCL2* mutations observed in our study (1.5–49.3%) and the absence of *BCL2* mutations at SC2 in three out of seven patients indicate that acquired changes other than mutations in BCL2 also contribute to drug resistance.

Previous studies revealed that overexpression of WT or mutated BCL2 in CLL cells or cell lines could increase their resistance to the cytotoxic effects of venetoclax [15, 48]. Similarly, we found that MEC1 or MEC1-ROR1 cells transfected to express high levels of *BCL2*^*WT*^, *BCL2*^*A113P*^, or *BCL2*^*G101V*^ also had reduced sensitivity to venetoclax compared to the parent MEC1 or MEC1-ROR1 cells. Despite the noted resistance of MEC1 cells to venetoclax [45], we still found that MEC1-ROR1 were even less sensitive to this drug, indicating that the expression of ROR1 per se can enhance drug resistance.

Previous studies showed that leukemia microenvironment could inhibit the capacity of venetoclax to induce CLL-cell apoptosis [15, 18]. Moreover, CLL cells of refractory patients appeared more resistant to venetoclax when cultured with accessory cells found within the leukemia microenvironment [15, 18]. Wnt5a, which may be provided by such accessory cells [19], can induce ROR1-signaling, which this and prior studies show enhance expression of ERK1/2 and NF-κB target genes [19, 21]. Moreover, treatment of ROR1^Hi^ CLL cells with Wnt5a increased expression of BCL-XL and enhanced the resistance to venetoclax in CLL cells. Treatment of the CLL with the inhibitory anti-ROR1 mAb zilovertamab, on the other hand, inhibited Wnt5a-induced increases in BCL-XL and resistance to venetoclax, indicating that this effect of Wnt5a is mediated via ROR1.

Collectively, these studies indicate that expression of ROR1 and ROR1-signaling may reduce the sensitivity of leukemia cells to venetoclax, potentially helping to contribute to venetoclax resistance. We hypothesize that strategies targeting ROR1 may enhance the efficacy of venetoclax-based regimens and/or help mitigate the risk of acquiring resistance to venetoclax therapy.

## Supplementary information

Table S1

Table S2

Table S3

Table S4

Supplemental Figure Legends

Supplemental Figures
