# Supplementary material for: High expression level of ROR1 and ROR1-signaling associates with venetoclax resistance in chronic lymphocytic leukemia
Source: Leukemia. 2022 Apr 13;36(6):1609–18. doi: 10.1038/s41375-022-01543-y (PMC9162914; doi:10.1038/s41375-022-01543-y)
Supplement: Supplementary file 1 — Table S1 [file 41375_2022_1543_MOESM1_ESM.docx]

| Gene Sets | SIZE | ES | NES | NOM p-val | FDR q-val |
| --- | --- | --- | --- | --- | --- |
| NF-κB JASPAR PREDICTED TRANSCRIPTION FACTORS TARGETS (37, 42) | 3077 | 0.05 | 3.11 | 0.000 | 0.000 |
| NF-κB TARGET GENES (40) | 60 | 0.22 | 1.95 | 0.000 | 0.013 |
| GO_NON_CANONICAL_WNT_SIGNALING_PATHWAY (38) | 133 | 0.13 | 1.68 | 0.031 | 0.046 |
| NF-κB TARGET GENES (41) | 84 | 0.14 | 1.41 | 0.098 | 0.146 |
| GO_CANONICAL_WNT_SIGNALING_PATHWAY (38) | 85 | 0.09 | 0.97 | 0.460 | 0.517 |

**Table S1.** GSEA of the genes expressed in negatively-selected CLL cells collected at MRD progression on venetoclax therapy (SC2) versus those expressed by the negatively-selected CLL cells collected from the same patients prior to treatment (SC1). GSEA on the transcriptomes of CLL cells collected at SC2 versus SC1, evaluating for differences in the expression of NF-κB target genes and of genes induced by canonical or non-canonical Wnt signaling pathway.(37, 38, 40-42) Gene-set size (SIZE), enrichment score (ES), normalized ES (NES), nominal p value (NOM p-val), and FDR q value (FDR q) are indicated.
