# Supplementary material for: High expression level of ROR1 and ROR1-signaling associates with venetoclax resistance in chronic lymphocytic leukemia
Source: Leukemia. 2022 Apr 13;36(6):1609–18. doi: 10.1038/s41375-022-01543-y (PMC9162914; doi:10.1038/s41375-022-01543-y)
Supplement: Supplementary file 2 — Table S2 [file 41375_2022_1543_MOESM2_ESM.docx]

**Table S2.** Significantly differentially expressed genes in negatively-selected CLL cells collected at MRD progression on venetoclax therapy (SC2) versus those expressed by the negatively-selected CLL cells collected from the same patients prior to treatment (SC1) with log2 fold change greater than 1.2 or less than -1.2. Gene Name, Fold change, log2 fold change and p-values are indicated.

| Gene Name | Fold Change | Log Fold Change | p-Value |
| --- | --- | --- | --- |
| SAC3D1 | 12.62 | 3.66 | 0.0000001 |
| CNR1 | -9.91 | -3.31 | 0.0000002 |
| TST | 12.48 | 3.64 | 0.0000005 |
| C3AR1 | 9.15 | 3.19 | 0.0000063 |
| VMO1 | 11.69 | 3.55 | 0.0000068 |
| HOXB4 | 7.16 | 2.84 | 0.0000074 |
| TCN2 | 8.46 | 3.08 | 0.0000076 |
| SDSL | 11.00 | 3.46 | 0.0000078 |
| SLC25A20 | 6.26 | 2.65 | 0.0000137 |
| ALDH3B1 | 5.14 | 2.36 | 0.0000153 |
| RRM2 | 8.21 | 3.04 | 0.0000222 |
| EPDR1 | 9.86 | 3.30 | 0.0000268 |
| EZR | -2.84 | -1.51 | 0.0000299 |
| CES1 | 9.06 | 3.18 | 0.0000339 |
| TNFSF10 | 4.18 | 2.06 | 0.0000384 |
| EXTL2 | 3.48 | 1.80 | 0.0000408 |
| HSD17B2 | -8.78 | -3.13 | 0.0000410 |
| NPY1R | -9.06 | -3.18 | 0.0000511 |
| ENDOG | 4.34 | 2.12 | 0.0000543 |
| CFD | 7.15 | 2.84 | 0.0000559 |
| LAMP5 | 8.45 | 3.08 | 0.0000640 |
| LIPC | 8.17 | 3.03 | 0.0000654 |
| GZMA | 7.56 | 2.92 | 0.0000783 |
| PMEL | 5.07 | 2.34 | 0.0000841 |
| LRRC75B | 3.33 | 1.74 | 0.0000870 |
| TNFAIP8L2 | 6.37 | 2.67 | 0.0000878 |
| MIR4435-2HG | 6.20 | 2.63 | 0.0000878 |
| GIMAP6 | 7.54 | 2.92 | 0.0000897 |
| N6AMT2 | 4.72 | 2.24 | 0.0000961 |
| TMEM176B | 7.79 | 2.96 | 0.0001030 |
| LRRC25 | 5.16 | 2.37 | 0.0001190 |
| NUCB2 | 6.10 | 2.61 | 0.0001220 |
| SDHAF3 | 3.55 | 1.83 | 0.0001220 |
| LOC100129055 | -7.73 | -2.95 | 0.0001300 |
| AKR1C3 | 7.97 | 2.99 | 0.0001380 |
| TKTL1 | 6.88 | 2.78 | 0.0001530 |
| CNP | 3.37 | 1.75 | 0.0001550 |
| FCGR3A | 7.48 | 2.90 | 0.0001590 |
| KIF23 | 5.90 | 2.56 | 0.0001610 |
| ARID5A | -2.70 | -1.43 | 0.0001730 |
| FMO4 | 3.45 | 1.78 | 0.0001740 |
| PIGZ | 4.09 | 2.03 | 0.0001770 |
| LILRA1 | 7.18 | 2.84 | 0.0001780 |
| MAPK12 | 5.22 | 2.39 | 0.0001860 |
| PPP1R14C | 6.77 | 2.76 | 0.0001890 |
| MATN1-AS1 | -3.61 | -1.85 | 0.0001890 |
| CCR1 | 7.16 | 2.84 | 0.0001970 |
| ANKRD53 | -4.71 | -2.24 | 0.0002020 |
| NKG7 | 6.64 | 2.73 | 0.0002030 |
| PYGL | 6.34 | 2.66 | 0.0002110 |
| KLRG1 | 5.91 | 2.56 | 0.0002120 |
| ZNF268 | -2.63 | -1.39 | 0.0002140 |
| PRF1 | 6.32 | 2.66 | 0.0002220 |
| CLIC3 | 6.28 | 2.65 | 0.0002260 |
| MORN4 | 4.66 | 2.22 | 0.0002320 |
| EXOSC5 | 3.39 | 1.76 | 0.0002370 |
| BCKDK | 3.16 | 1.66 | 0.0002370 |
| CX3CR1 | 7.31 | 2.87 | 0.0002380 |
| SLC47A1 | 6.62 | 2.73 | 0.0002400 |
| S100A11 | 4.36 | 2.12 | 0.0002440 |
| NAP1L2 | 6.07 | 2.60 | 0.0002450 |
| FGL2 | 6.73 | 2.75 | 0.0002470 |
| CD300LF | 6.20 | 2.63 | 0.0002480 |
| C9orf69 | 4.35 | 2.12 | 0.0002700 |
| AK5 | 6.48 | 2.70 | 0.0002720 |
| MS4A7 | 5.82 | 2.54 | 0.0002720 |
| EIF2B3 | 2.46 | 1.30 | 0.0002730 |
| 3-Sep | 5.81 | 2.54 | 0.0002760 |
| SMPDL3A | 7.23 | 2.85 | 0.0002870 |
| GZMB | 6.05 | 2.60 | 0.0002870 |
| LINC00877 | 7.03 | 2.81 | 0.0002910 |
| LRRC4 | 5.94 | 2.57 | 0.0002940 |
| TMEM254 | 3.39 | 1.76 | 0.0002950 |
| SPC25 | 7.26 | 2.86 | 0.0003030 |
| PAQR4 | 4.46 | 2.16 | 0.0003060 |
| ARL6IP5 | 2.66 | 1.41 | 0.0003060 |
| NPL | 4.51 | 2.17 | 0.0003120 |
| C16orf59 | 5.31 | 2.41 | 0.0003240 |
| ZNF426 | -2.34 | -1.23 | 0.0003320 |
| VNN1 | 6.85 | 2.78 | 0.0003400 |
| FGFBP2 | 6.37 | 2.67 | 0.0003460 |
| HMBS | 3.73 | 1.90 | 0.0003660 |
| SPTSSB | 7.00 | 2.81 | 0.0003700 |
| LMO2 | 6.23 | 2.64 | 0.0003760 |
| KLRB1 | 6.08 | 2.60 | 0.0003820 |
| RPS6KA1 | 2.76 | 1.47 | 0.0003870 |
| ALDH1A1 | 6.37 | 2.67 | 0.0003890 |
| CCR2 | 5.03 | 2.33 | 0.0003940 |
| IGFBP7 | 5.97 | 2.58 | 0.0004100 |
| HRSP12 | 3.98 | 1.99 | 0.0004100 |
| NUDT1 | 3.60 | 1.85 | 0.0004100 |
| AQP9 | 7.16 | 2.84 | 0.0004160 |
| CTSW | 5.61 | 2.49 | 0.0004250 |
| LAMB2 | -4.52 | -2.18 | 0.0004310 |
| C5AR1 | 5.19 | 2.38 | 0.0004410 |
| GZMH | 5.50 | 2.46 | 0.0004560 |
| AURKB | 5.89 | 2.56 | 0.0004630 |
| ITGB2 | 4.45 | 2.15 | 0.0004770 |
| CST7 | 5.58 | 2.48 | 0.0004780 |
| MLC1 | 6.51 | 2.70 | 0.0004840 |
| CHEK2 | 2.75 | 1.46 | 0.0004870 |
| CDCA7 | 4.56 | 2.19 | 0.0004980 |
| NUDT16P1 | 6.62 | 2.73 | 0.0005000 |
| FAH | 4.48 | 2.16 | 0.0005040 |
| SLC29A1 | 3.97 | 1.99 | 0.0005090 |
| CDK5 | 2.97 | 1.57 | 0.0005090 |
| C1orf233 | 3.50 | 1.81 | 0.0005110 |
| GPR162 | 6.90 | 2.79 | 0.0005120 |
| AOAH | 4.68 | 2.23 | 0.0005160 |
| GIMAP7 | 6.35 | 2.67 | 0.0005200 |
| SLC31A2 | 5.46 | 2.45 | 0.0005220 |
| SLAMF8 | 6.83 | 2.77 | 0.0005290 |
| AFMID | 2.75 | 1.46 | 0.0005350 |
| TSPAN2 | 6.20 | 2.63 | 0.0005380 |
| DPAGT1 | 2.62 | 1.39 | 0.0005640 |
| CDCA8 | 4.94 | 2.31 | 0.0006010 |
| RPP25 | 3.69 | 1.88 | 0.0006020 |
| MT1F | 3.76 | 1.91 | 0.0006050 |
| CD300LB | 3.83 | 1.94 | 0.0006080 |
| DHCR24 | 3.50 | 1.81 | 0.0006110 |
| CD300E | 6.31 | 2.66 | 0.0006140 |
| SECTM1 | 6.53 | 2.71 | 0.0006180 |
| SERPINA1 | 5.94 | 2.57 | 0.0006180 |
| ADAP1 | 3.95 | 1.98 | 0.0006220 |
| GLIPR2 | 3.78 | 1.92 | 0.0006240 |
| RGS3 | 4.01 | 2.00 | 0.0006280 |
| CD300A | 5.33 | 2.41 | 0.0006290 |
| SLC4A3 | 5.36 | 2.42 | 0.0006330 |
| JCHAIN | 6.01 | 2.59 | 0.0006530 |
| CHRNB1 | 3.53 | 1.82 | 0.0006800 |
| LILRA5 | 6.31 | 2.66 | 0.0006840 |
| DFFB | 2.35 | 1.23 | 0.0007100 |
| LOC152225 | 6.40 | 2.68 | 0.0007230 |
| HN1L | 2.70 | 1.43 | 0.0007240 |
| HMOX1 | 4.01 | 2.00 | 0.0007360 |
| FAM129B | 5.72 | 2.52 | 0.0007400 |
| DNASE1L3 | 5.52 | 2.46 | 0.0007420 |
| LPAR6 | 5.10 | 2.35 | 0.0007450 |
| UNC45A | 2.49 | 1.32 | 0.0007450 |
| CAT | 3.36 | 1.75 | 0.0007620 |
| LRRN4CL | -5.92 | -2.57 | 0.0007640 |
| CYP4F22 | 6.46 | 2.69 | 0.0007860 |
| EPB41L3 | 5.22 | 2.38 | 0.0007940 |
| BFSP1 | 5.57 | 2.48 | 0.0008100 |
| DNMT3B | 4.60 | 2.20 | 0.0008170 |
| MCM3 | 2.40 | 1.26 | 0.0008200 |
| MYOF | 5.52 | 2.46 | 0.0008400 |
| SERPINF2 | 3.62 | 1.86 | 0.0008800 |
| PSMG3-AS1 | 2.55 | 1.35 | 0.0009320 |
| CUEDC1 | 5.42 | 2.44 | 0.0009330 |
| NACC2 | 4.41 | 2.14 | 0.0009370 |
| KIFC1 | 4.80 | 2.26 | 0.0009410 |
| ADORA2B | 6.22 | 2.64 | 0.0009520 |
| SH2D1B | 5.85 | 2.55 | 0.0009630 |
| ACRBP | 2.75 | 1.46 | 0.0009740 |
| APOL2 | 2.34 | 1.23 | 0.0010020 |
| GSN | 3.62 | 1.86 | 0.0010060 |
| CPPED1 | 2.62 | 1.39 | 0.0010080 |
| ETS2 | 4.89 | 2.29 | 0.0010220 |
| SMKR1 | 4.24 | 2.08 | 0.0010310 |
| C10orf11 | 6.26 | 2.65 | 0.0010350 |
| ZDHHC16 | 2.48 | 1.31 | 0.0010350 |
| FBP1 | 4.13 | 2.05 | 0.0010370 |
| GIMAP4 | 5.98 | 2.58 | 0.0010400 |
| NRIP3 | 5.68 | 2.51 | 0.0010530 |
| MT2A | 3.90 | 1.96 | 0.0010850 |
| DTYMK | 2.97 | 1.57 | 0.0010870 |
| IDH2 | 2.69 | 1.43 | 0.0010950 |
| HJURP | 4.71 | 2.24 | 0.0011060 |
| PSPH | 2.57 | 1.36 | 0.0011160 |
| FOXM1 | 3.78 | 1.92 | 0.0011260 |
| MELK | 4.24 | 2.08 | 0.0011500 |
| MON1A | 3.52 | 1.82 | 0.0011560 |
| LY6G5C | 4.01 | 2.00 | 0.0011670 |
| FBLN5 | 5.81 | 2.54 | 0.0011750 |
| AMPH | -5.51 | -2.46 | 0.0012050 |
| CTSL | 6.12 | 2.61 | 0.0012070 |
| PSD2 | -2.99 | -1.58 | 0.0012110 |
| GZMK | 5.50 | 2.46 | 0.0012310 |
| GABARAPL1 | -2.54 | -1.34 | 0.0012350 |
| PTPN18 | 2.56 | 1.35 | 0.0012430 |
| SAMHD1 | 4.28 | 2.10 | 0.0012440 |
| CD7 | 4.73 | 2.24 | 0.0012470 |
| PPP2R4 | 2.51 | 1.33 | 0.0012560 |
| C12orf79 | -4.10 | -2.04 | 0.0012600 |
| YIF1B | 2.72 | 1.44 | 0.0012650 |
| TMIGD2 | 5.56 | 2.47 | 0.0012740 |
| TMEM109 | 2.95 | 1.56 | 0.0012740 |
| GZMM | 4.77 | 2.25 | 0.0013260 |
| ENHO | 5.39 | 2.43 | 0.0013320 |
| NHLH1 | -3.82 | -1.93 | 0.0013430 |
| DMXL2 | 4.84 | 2.28 | 0.0013690 |
| KCNMB1 | 5.75 | 2.52 | 0.0013720 |
| MATK | 4.66 | 2.22 | 0.0013960 |
| ZNF185 | 2.83 | 1.50 | 0.0013980 |
| CXCR1 | 5.84 | 2.55 | 0.0014110 |
| FAM110A | 2.79 | 1.48 | 0.0014110 |
| MVK | 2.70 | 1.43 | 0.0014400 |
| KIR3DL2 | 5.36 | 2.42 | 0.0014730 |
| COL15A1 | 5.14 | 2.36 | 0.0015090 |
| MPST | 3.74 | 1.90 | 0.0015240 |
| COMT | 2.68 | 1.42 | 0.0015350 |
| SLC24A4 | 5.29 | 2.40 | 0.0015370 |
| CFB | -3.79 | -1.92 | 0.0015390 |
| IER5L | 4.72 | 2.24 | 0.0015660 |
| SLC37A4 | 2.89 | 1.53 | 0.0015660 |
| LDLRAD3 | 4.58 | 2.19 | 0.0015780 |
| ADCK1 | 2.30 | 1.20 | 0.0016000 |
| PTGER2 | 5.31 | 2.41 | 0.0016030 |
| NOTCH4 | 2.74 | 1.45 | 0.0016060 |
| ALDH4A1 | 2.85 | 1.51 | 0.0016370 |
| CEBPA | 4.34 | 2.12 | 0.0016570 |
| DNASE2 | 2.95 | 1.56 | 0.0016860 |
| ICMT | 2.78 | 1.48 | 0.0016900 |
| CPVL | 4.82 | 2.27 | 0.0016920 |
| RALBP1 | 2.84 | 1.51 | 0.0017190 |
| H2AFY2 | 5.61 | 2.49 | 0.0017230 |
| CEP55 | 4.44 | 2.15 | 0.0017280 |
| BEND6 | 5.55 | 2.47 | 0.0017340 |
| GNLY | 4.85 | 2.28 | 0.0017410 |
| LXN | 3.61 | 1.85 | 0.0017440 |
| ITGA6 | 4.83 | 2.27 | 0.0017730 |
| SLC1A4 | -2.71 | -1.44 | 0.0017730 |
| NLRC4 | 4.91 | 2.30 | 0.0018060 |
| ASB2 | 3.79 | 1.92 | 0.0018070 |
| LOC257396 | 4.92 | 2.30 | 0.0018140 |
| GLRX | 4.26 | 2.09 | 0.0018370 |
| PPT2 | 4.02 | 2.01 | 0.0018430 |
| LOC645166 | 4.62 | 2.21 | 0.0018610 |
| PKMYT1 | 5.23 | 2.39 | 0.0018730 |
| GCNT1 | 4.87 | 2.28 | 0.0018900 |
| SIGLEC7 | 5.37 | 2.42 | 0.0019110 |
| AURKA | 4.26 | 2.09 | 0.0019130 |
| TSPAN17 | 2.40 | 1.26 | 0.0019240 |
| IL32 | 4.66 | 2.22 | 0.0019270 |
| LOC100507195 | 4.30 | 2.11 | 0.0019290 |
| SIRPB1 | 4.85 | 2.28 | 0.0019400 |
| CBR1 | 4.19 | 2.07 | 0.0019400 |
| TM6SF1 | 4.39 | 2.13 | 0.0019460 |
| CAV1 | 5.27 | 2.40 | 0.0019560 |
| SMPD3 | 3.75 | 1.91 | 0.0019680 |
| ELOVL6 | 4.01 | 2.00 | 0.0019710 |
| S100A3 | 5.64 | 2.50 | 0.0019830 |
| OAS2 | 2.33 | 1.22 | 0.0019950 |
| ABHD6 | 3.43 | 1.78 | 0.0019990 |
| ADGRG5 | 4.86 | 2.28 | 0.0020150 |
| ADGRE3 | 5.63 | 2.49 | 0.0020360 |
| IFITM3 | 3.89 | 1.96 | 0.0020410 |
| THAP8 | 3.50 | 1.81 | 0.0020570 |
| HBZ | -5.12 | -2.36 | 0.0020810 |
| CDH23 | 4.28 | 2.10 | 0.0021010 |
| LOC100506585 | 5.49 | 2.46 | 0.0021070 |
| CD160 | 4.94 | 2.30 | 0.0021270 |
| GNAQ | 4.51 | 2.17 | 0.0021340 |
| CAMK1 | 4.85 | 2.28 | 0.0021490 |
| CCNB1 | 3.52 | 1.81 | 0.0021560 |
| C12orf75 | 4.39 | 2.13 | 0.0021810 |
| PNPO | 3.48 | 1.80 | 0.0021890 |
| SRXN1 | 4.16 | 2.06 | 0.0021940 |
| HK3 | 5.14 | 2.36 | 0.0022000 |
| SAA2 | -4.02 | -2.01 | 0.0022220 |
| SNX9 | -2.68 | -1.42 | 0.0022370 |
| LINC00152 | 3.30 | 1.72 | 0.0022540 |
| ARMC10 | 2.54 | 1.34 | 0.0022720 |
| CLEC7A | 4.35 | 2.12 | 0.0022790 |
| MOXD1 | 4.82 | 2.27 | 0.0022820 |
| COPZ2 | 4.07 | 2.03 | 0.0023000 |
| PRPSAP1 | 2.88 | 1.53 | 0.0023850 |
| KLRD1 | 4.62 | 2.21 | 0.0024090 |
| TTC38 | 3.56 | 1.83 | 0.0024400 |
| CALHM2 | 2.93 | 1.55 | 0.0024740 |
| TSPAN4 | 3.55 | 1.83 | 0.0024850 |
| SPR | 5.13 | 2.36 | 0.0025010 |
| LILRA3 | 5.40 | 2.43 | 0.0025020 |
| FAM20C | 4.10 | 2.04 | 0.0025170 |
| SHMT1 | 2.42 | 1.28 | 0.0025300 |
| PRDX3 | 2.74 | 1.46 | 0.0025400 |
| RDM1 | 5.34 | 2.42 | 0.0025450 |
| AIM2 | 2.48 | 1.31 | 0.0025710 |
| RUVBL1 | 3.10 | 1.63 | 0.0026390 |
| CCDC28B | 3.14 | 1.65 | 0.0026530 |
| KIR3DL1 | 5.20 | 2.38 | 0.0026600 |
| CTBP2 | 4.46 | 2.16 | 0.0026730 |
| LINC00861 | 4.32 | 2.11 | 0.0026960 |
| MS4A14 | 4.67 | 2.22 | 0.0027230 |
| DRD3 | -5.38 | -2.43 | 0.0027230 |
| HOPX | 4.76 | 2.25 | 0.0027340 |
| CCL5 | 4.21 | 2.08 | 0.0027800 |
| PGCP1 | -5.05 | -2.34 | 0.0028260 |
| EEPD1 | 4.82 | 2.27 | 0.0028290 |
| OSER1-AS1 | 3.53 | 1.82 | 0.0028540 |
| LRP2BP | -3.23 | -1.69 | 0.0028600 |
| AGTRAP | 3.38 | 1.76 | 0.0028790 |
| SLCO4A1 | -3.05 | -1.61 | 0.0028910 |
| CSF1R | 5.30 | 2.41 | 0.0028920 |
| IFIT1 | 4.35 | 2.12 | 0.0029260 |
| NR1H3 | 3.34 | 1.74 | 0.0029410 |
| TMEM229B | 4.39 | 2.13 | 0.0029650 |
| HEBP1 | 3.94 | 1.98 | 0.0029650 |
| PRR5L | 4.87 | 2.28 | 0.0029790 |
| IGFBP6 | 5.16 | 2.37 | 0.0030100 |
| SNORD71 | -4.20 | -2.07 | 0.0030220 |
| TMEM2 | -2.78 | -1.48 | 0.0030260 |
| UPP1 | 3.62 | 1.86 | 0.0030430 |
| LILRB3 | 4.01 | 2.00 | 0.0030650 |
| CCNB2 | 3.45 | 1.79 | 0.0030700 |
| PCDHGC5 | -5.18 | -2.37 | 0.0030860 |
| SLC9A7P1 | 5.16 | 2.37 | 0.0030910 |
| NAT8L | 4.39 | 2.14 | 0.0031140 |
| IL6R | 3.63 | 1.86 | 0.0031980 |
| KIF4A | 4.54 | 2.18 | 0.0032120 |
| ADGRG1 | 4.56 | 2.19 | 0.0033060 |
| PIK3R6 | 4.05 | 2.02 | 0.0033280 |
| VPS26B | 2.78 | 1.47 | 0.0033280 |
| TCAIM | 2.62 | 1.39 | 0.0033330 |
| PRKAR1B | 2.56 | 1.36 | 0.0033330 |
| FES | 3.19 | 1.68 | 0.0033620 |
| TMEM256 | 2.37 | 1.24 | 0.0034240 |
| PADI6 | 4.83 | 2.27 | 0.0034320 |
| ADGRE1 | 4.53 | 2.18 | 0.0034470 |
| RTN4RL2 | 4.36 | 2.12 | 0.0034490 |
| E2F1 | 2.53 | 1.34 | 0.0034520 |
| TNFRSF25 | 3.40 | 1.76 | 0.0034820 |
| CD4 | 4.14 | 2.05 | 0.0034830 |
| SOCS2 | 4.58 | 2.20 | 0.0034840 |
| FAM118B | 2.82 | 1.50 | 0.0035040 |
| C1GALT1C1 | 2.99 | 1.58 | 0.0035590 |
| BPNT1 | 2.51 | 1.33 | 0.0036250 |
| TAF7L | -4.10 | -2.03 | 0.0036880 |
| APMAP | 3.07 | 1.62 | 0.0037080 |
| DAPK1 | 4.20 | 2.07 | 0.0037230 |
| SPAG4 | -3.90 | -1.96 | 0.0037310 |
| KCNE3 | 5.09 | 2.35 | 0.0037400 |
| TYMP | 2.71 | 1.44 | 0.0037550 |
| HAVCR2 | 3.45 | 1.78 | 0.0038050 |
| VANGL1 | 4.18 | 2.06 | 0.0038420 |
| MFNG | 2.38 | 1.25 | 0.0038460 |
| CDCA3 | 3.90 | 1.97 | 0.0038480 |
| THEM6 | 2.83 | 1.50 | 0.0039040 |
| ANKRD50 | 4.64 | 2.21 | 0.0039220 |
| SERPINE2 | 3.93 | 1.98 | 0.0039390 |
| METRN | 3.58 | 1.84 | 0.0039500 |
| C1orf61 | 3.92 | 1.97 | 0.0039600 |
| C11orf96 | 4.75 | 2.25 | 0.0039820 |
| RAMP1 | 4.82 | 2.27 | 0.0040230 |
| LILRA6 | 4.90 | 2.29 | 0.0040340 |
| DUSP7 | 2.88 | 1.53 | 0.0040350 |
| ID2 | 4.00 | 2.00 | 0.0040450 |
| CDT1 | 3.64 | 1.86 | 0.0040840 |
| HES6 | 3.44 | 1.78 | 0.0041080 |
| ACSM3 | -2.79 | -1.48 | 0.0041210 |
| C19orf38 | 3.10 | 1.63 | 0.0041250 |
| GAB2 | -3.14 | -1.65 | 0.0041290 |
| PTGDS | 3.98 | 1.99 | 0.0041360 |
| NFE2 | 4.42 | 2.15 | 0.0041390 |
| KIR2DL3 | 4.72 | 2.24 | 0.0041510 |
| MRS2 | 2.47 | 1.31 | 0.0041550 |
| C1orf21 | 4.53 | 2.18 | 0.0041870 |
| FAM105A | 3.92 | 1.97 | 0.0042120 |
| CD247 | 4.00 | 2.00 | 0.0042130 |
| TXK | 3.32 | 1.73 | 0.0043100 |
| UBE2C | 3.19 | 1.67 | 0.0043700 |
| P2RY2 | 4.84 | 2.28 | 0.0043750 |
| LOC143666 | -2.88 | -1.53 | 0.0043860 |
| BIRC5 | 3.81 | 1.93 | 0.0044000 |
| HDHD2 | 2.36 | 1.24 | 0.0044110 |
| CMKLR1 | 4.89 | 2.29 | 0.0044670 |
| CISD1 | 2.50 | 1.32 | 0.0044690 |
| MT1G | 4.88 | 2.29 | 0.0045050 |
| C4orf27 | 3.13 | 1.65 | 0.0045190 |
| LINC01272 | 4.91 | 2.30 | 0.0045730 |
| KIR2DL4 | 4.64 | 2.21 | 0.0045850 |
| PIGF | 2.51 | 1.33 | 0.0046250 |
| SAMD3 | 4.24 | 2.08 | 0.0047110 |
| SYTL3 | 3.08 | 1.62 | 0.0047270 |
| MSLN | 4.66 | 2.22 | 0.0047430 |
| DPF1 | 4.22 | 2.08 | 0.0047450 |
| RAB6B | 4.88 | 2.29 | 0.0047690 |
| SYNGR3 | -2.85 | -1.51 | 0.0047770 |
| HOXB6 | 4.88 | 2.29 | 0.0047780 |
| SLC1A7 | 4.46 | 2.16 | 0.0047780 |
| SPON2 | 3.83 | 1.94 | 0.0048220 |
| MED11 | 2.45 | 1.29 | 0.0048390 |
| CAMKK2 | 2.44 | 1.28 | 0.0049360 |
| CMC1 | 3.32 | 1.73 | 0.0049560 |
| PLA2G16 | 3.54 | 1.82 | 0.0049820 |
| TNS2 | -3.72 | -1.89 | 0.0050010 |
| C7orf72 | 3.82 | 1.93 | 0.0050190 |
| STRIP2 | 3.56 | 1.83 | 0.0050230 |
| CDKN2C | 3.01 | 1.59 | 0.0050340 |
| HES1 | 4.64 | 2.21 | 0.0050540 |
| LAT | 3.07 | 1.62 | 0.0050560 |
| SPATA17 | 4.78 | 2.26 | 0.0050640 |
| FUT7 | 4.82 | 2.27 | 0.0050900 |
| MAP7 | 3.80 | 1.93 | 0.0050990 |
| KANK3 | -3.64 | -1.86 | 0.0051070 |
| IL12RB2 | 4.52 | 2.18 | 0.0051420 |
| C17orf62 | 2.44 | 1.29 | 0.0051620 |
| SMAD1 | 4.76 | 2.25 | 0.0051780 |
| SLC44A1 | 3.00 | 1.59 | 0.0051990 |
| CLEC12A | 4.18 | 2.06 | 0.0052090 |
| RNF157 | 3.45 | 1.79 | 0.0052420 |
| NFAM1 | 4.44 | 2.15 | 0.0052700 |
| IL1R2 | 4.53 | 2.18 | 0.0053030 |
| PPP1CA | 2.30 | 1.20 | 0.0053060 |
| IL17RC | 2.86 | 1.52 | 0.0053770 |
| CRABP2 | -2.77 | -1.47 | 0.0053840 |
| RENBP | 2.56 | 1.36 | 0.0054330 |
| AXL | 4.48 | 2.16 | 0.0054460 |
| ABI3 | 4.29 | 2.10 | 0.0054460 |
| ABHD17C | 2.77 | 1.47 | 0.0054900 |
| SIGLEC12 | 4.74 | 2.25 | 0.0055130 |
| C14orf142 | 2.46 | 1.30 | 0.0056310 |
| ARL2 | 2.78 | 1.47 | 0.0056500 |
| LGALS9B | 3.80 | 1.92 | 0.0056710 |
| CD244 | 4.08 | 2.03 | 0.0056970 |
| MARCO | 4.68 | 2.23 | 0.0057520 |
| GLCE | 2.39 | 1.26 | 0.0057990 |
| LOC100129550 | -2.52 | -1.34 | 0.0058390 |
| MGAT4A | 3.55 | 1.83 | 0.0058410 |
| TNFSF13B | 2.66 | 1.41 | 0.0058470 |
| DHRS13 | 2.42 | 1.27 | 0.0058530 |
| TCEB3-AS1 | 2.54 | 1.35 | 0.0058930 |
| SELPLG | 3.56 | 1.83 | 0.0058950 |
| TSSK4 | -3.75 | -1.91 | 0.0059030 |
| ALDH7A1 | 4.22 | 2.08 | 0.0059070 |
| MAP1B | -4.28 | -2.10 | 0.0059450 |
| C1QB | 4.67 | 2.22 | 0.0059760 |
| USP11 | -2.37 | -1.24 | 0.0059990 |
| PTGDR | 4.35 | 2.12 | 0.0060100 |
| VPS72 | 2.34 | 1.23 | 0.0060240 |
| C1orf53 | 3.32 | 1.73 | 0.0060250 |
| RPL23AP82 | 2.83 | 1.50 | 0.0060250 |
| C10orf54 | 2.89 | 1.53 | 0.0060280 |
| VKORC1 | 2.72 | 1.44 | 0.0060310 |
| PRSS30P | 4.49 | 2.17 | 0.0060400 |
| RHEBL1 | 2.89 | 1.53 | 0.0060440 |
| CD2 | 3.83 | 1.94 | 0.0060690 |
| CYTL1 | 3.60 | 1.85 | 0.0060710 |
| STOM | 3.20 | 1.68 | 0.0060810 |
| SIGLEC17P | 4.14 | 2.05 | 0.0060820 |
| KLRC3 | 4.00 | 2.00 | 0.0061040 |
| KLRC1 | 4.51 | 2.17 | 0.0061140 |
| MARVELD1 | 3.42 | 1.77 | 0.0061410 |
| FCRLB | 2.58 | 1.37 | 0.0061720 |
| HNMT | 4.63 | 2.21 | 0.0061960 |
| BATF | 2.98 | 1.58 | 0.0062100 |
| PRSS23 | 4.38 | 2.13 | 0.0062370 |
| TIE1 | 4.06 | 2.02 | 0.0062710 |
| ICOSLG | -2.65 | -1.40 | 0.0063200 |
| CTSD | 2.90 | 1.54 | 0.0064160 |
| LINC01260 | 4.24 | 2.08 | 0.0064480 |
| LAIR1 | 3.90 | 1.96 | 0.0064550 |
| IFI30 | 3.03 | 1.60 | 0.0064740 |
| TTC9B | -4.56 | -2.19 | 0.0065040 |
| ZNF286B | -2.45 | -1.29 | 0.0065270 |
| SNORA38B | -3.81 | -1.93 | 0.0065460 |
| FASLG | 4.14 | 2.05 | 0.0065470 |
| ASCL2 | 4.20 | 2.07 | 0.0065610 |
| CYP2J2 | 2.84 | 1.51 | 0.0065700 |
| MYCL | 4.21 | 2.07 | 0.0065770 |
| HOXB7 | 2.81 | 1.49 | 0.0066000 |
| SCARNA9L | -3.42 | -1.77 | 0.0066330 |
| ACOT4 | 3.78 | 1.92 | 0.0067760 |
| DKFZp779M0652 | 4.57 | 2.19 | 0.0068520 |
| STEAP3 | 4.14 | 2.05 | 0.0069050 |
| HIBADH | 2.61 | 1.38 | 0.0069230 |
| TMEM99 | 3.05 | 1.61 | 0.0069450 |
| FAM212A | 3.85 | 1.95 | 0.0069520 |
| B4GALT6 | 4.21 | 2.07 | 0.0069540 |
| ECE2 | 4.00 | 2.00 | 0.0070050 |
| FCN1 | 3.99 | 2.00 | 0.0070210 |
| DAB2 | 4.19 | 2.07 | 0.0070260 |
| TC2N | 3.57 | 1.84 | 0.0070580 |
| ACOT13 | 2.74 | 1.45 | 0.0070840 |
| STXBP1 | 4.20 | 2.07 | 0.0071010 |
| PLA2G4A | 4.18 | 2.06 | 0.0071130 |
| AK8 | 4.13 | 2.05 | 0.0071320 |
| GBP1P1 | 4.45 | 2.15 | 0.0071740 |
| PDE4C | -4.07 | -2.02 | 0.0071780 |
| HDGFRP3 | 4.18 | 2.06 | 0.0072330 |
| CXCR2P1 | 4.39 | 2.13 | 0.0072880 |
| PCTP | 2.87 | 1.52 | 0.0073130 |
| IL2RB | 3.50 | 1.81 | 0.0073380 |
| SH2D2A | 3.86 | 1.95 | 0.0073840 |
| TBC1D4 | 3.17 | 1.67 | 0.0074730 |
| TMEM14A | 2.31 | 1.21 | 0.0074810 |
| C16orf71 | -3.36 | -1.75 | 0.0075020 |
| DTHD1 | 4.19 | 2.07 | 0.0075430 |
| FCER1G | 3.82 | 1.93 | 0.0075440 |
| IL1R1 | 4.13 | 2.05 | 0.0075460 |
| AKAP5 | -3.12 | -1.64 | 0.0075480 |
| SMIM6 | 4.18 | 2.06 | 0.0075750 |
| SLC43A1 | 2.78 | 1.48 | 0.0075870 |
| IFT81 | 2.55 | 1.35 | 0.0075980 |
| MGLL | 3.45 | 1.79 | 0.0076630 |
| CCR5 | 4.31 | 2.11 | 0.0076800 |
| LBH | 3.24 | 1.70 | 0.0076900 |
| CNDP1 | -3.16 | -1.66 | 0.0077280 |
| PAQR8 | 3.67 | 1.87 | 0.0077900 |
| TBXAS1 | 3.38 | 1.75 | 0.0078230 |
| ADGRE2 | 3.69 | 1.89 | 0.0078900 |
| SLC34A1 | 4.44 | 2.15 | 0.0079100 |
| PIK3R3 | 3.87 | 1.95 | 0.0079120 |
| SNAPIN | 2.35 | 1.24 | 0.0079200 |
| ADGRA3 | 4.28 | 2.10 | 0.0079280 |
| IRS2 | -2.72 | -1.44 | 0.0079350 |
| HIST1H2AC | 2.89 | 1.53 | 0.0079760 |
| PRADC1 | 2.46 | 1.30 | 0.0079870 |
| S1PR5 | 3.63 | 1.86 | 0.0079920 |
| C16orf95 | 3.23 | 1.69 | 0.0079980 |
| ZMYM6NB | 2.78 | 1.48 | 0.0080160 |
| P3H3 | 4.24 | 2.08 | 0.0080250 |
| PCK1 | -4.42 | -2.15 | 0.0080540 |
| RETN | 4.40 | 2.14 | 0.0081060 |
| LMAN2L | 2.40 | 1.26 | 0.0081280 |
| LAIR2 | 3.91 | 1.97 | 0.0082980 |
| SIGLEC6 | 3.69 | 1.88 | 0.0083000 |
| MIR223 | 2.91 | 1.54 | 0.0083030 |
| NAGA | 3.05 | 1.61 | 0.0083290 |
| RNF217 | 3.92 | 1.97 | 0.0083400 |
| RASGRP4 | 3.67 | 1.87 | 0.0083630 |
| BOLA3 | 2.54 | 1.34 | 0.0083790 |
| SMURF1 | -2.81 | -1.49 | 0.0084470 |
| CACNA2D3 | 3.90 | 1.97 | 0.0084500 |
| AMACR | 3.10 | 1.63 | 0.0085950 |
| CNRIP1 | 4.35 | 2.12 | 0.0086150 |
| SSC4D | 4.31 | 2.11 | 0.0087100 |
| PTCRA | 3.67 | 1.87 | 0.0087580 |
| MS4A4A | 4.11 | 2.04 | 0.0087930 |
| ZNF792 | 2.49 | 1.31 | 0.0088760 |
| CASC21 | 4.35 | 2.12 | 0.0088860 |
| KCNJ14 | -2.57 | -1.36 | 0.0088860 |
| CDKN1C | 3.54 | 1.82 | 0.0089070 |
| GNB3 | 4.16 | 2.06 | 0.0089890 |
| LZTS3 | -3.20 | -1.68 | 0.0090440 |
| BCL2L1 | 2.37 | 1.24 | 0.0091380 |
| PCSK5 | 4.04 | 2.01 | 0.0091980 |
| TYMS | 2.76 | 1.46 | 0.0092100 |
| FCRL6 | 4.10 | 2.04 | 0.0092450 |
| DHRS3 | 3.43 | 1.78 | 0.0092950 |
| ICAM2 | 2.60 | 1.38 | 0.0093120 |
| ARHGAP23 | -3.84 | -1.94 | 0.0093120 |
| SLC38A5 | 2.54 | 1.35 | 0.0093960 |
| ST8SIA6 | 3.81 | 1.93 | 0.0094590 |
| CASP10 | 2.92 | 1.55 | 0.0094800 |
| GRIK3 | -4.07 | -2.02 | 0.0094990 |
| TROAP | 2.91 | 1.54 | 0.0095400 |
| LPCAT2 | 3.80 | 1.92 | 0.0095850 |
| TPPP3 | 3.93 | 1.98 | 0.0096010 |
| TMC5 | 4.17 | 2.06 | 0.0096200 |
| TNFRSF11A | 3.81 | 1.93 | 0.0096430 |
| METTL7B | 4.24 | 2.08 | 0.0096660 |
| LSM10 | 2.52 | 1.33 | 0.0096990 |
| ELAVL3 | -3.85 | -1.94 | 0.0098280 |
| RPH3A | 4.26 | 2.09 | 0.0098600 |
| GAS1 | 4.15 | 2.05 | 0.0100300 |
| ANXA1 | 3.62 | 1.86 | 0.0101260 |
| ISOC2 | 2.71 | 1.44 | 0.0101530 |
| KIF2C | 3.19 | 1.67 | 0.0101790 |
| RASSF4 | 3.09 | 1.63 | 0.0102400 |
| SOCS6 | 3.29 | 1.72 | 0.0102840 |
| UNG | 2.77 | 1.47 | 0.0102860 |
| PCDH1 | 3.85 | 1.94 | 0.0103590 |
| TEX14 | -3.60 | -1.85 | 0.0103840 |
| ARHGEF37 | 3.78 | 1.92 | 0.0104190 |
| HES4 | 3.72 | 1.89 | 0.0104410 |
| ABCC3 | 3.89 | 1.96 | 0.0104440 |
| ZNF208 | -4.19 | -2.07 | 0.0105070 |
| NCALD | 3.90 | 1.97 | 0.0105110 |
| FLJ42351 | 3.00 | 1.58 | 0.0105190 |
| HSD3B7 | 3.14 | 1.65 | 0.0105210 |
| CXCR2 | 4.14 | 2.05 | 0.0106550 |
| PDGFC | 4.06 | 2.02 | 0.0107130 |
| SH2D1A | 3.47 | 1.80 | 0.0107390 |
| MCTP1 | 3.81 | 1.93 | 0.0107590 |
| RAB32 | 3.27 | 1.71 | 0.0108110 |
| NUP210L | -3.53 | -1.82 | 0.0108210 |
| DPPA4 | 3.92 | 1.97 | 0.0108560 |
| ARSJ | 4.18 | 2.06 | 0.0108620 |
| HTRA4 | -3.18 | -1.67 | 0.0108960 |
| ZDHHC14 | 3.27 | 1.71 | 0.0109060 |
| RGS9 | 3.73 | 1.90 | 0.0109180 |
| TMPRSS3 | 4.17 | 2.06 | 0.0109420 |
| SCARB1 | 3.70 | 1.89 | 0.0109650 |
| LRRC43 | 3.72 | 1.89 | 0.0109780 |
| C6orf132 | 4.17 | 2.06 | 0.0109800 |
| TNN | -4.08 | -2.03 | 0.0109860 |
| FPR2 | 4.10 | 2.04 | 0.0111140 |
| SORD | 2.55 | 1.35 | 0.0111640 |
| HGF | 4.14 | 2.05 | 0.0111790 |
| ITGAD | 3.44 | 1.78 | 0.0112510 |
| PMVK | 2.61 | 1.38 | 0.0112860 |
| CD9 | 3.39 | 1.76 | 0.0113200 |
| SLC6A4 | 3.73 | 1.90 | 0.0113220 |
| FAM129A | 3.77 | 1.91 | 0.0113860 |
| NPW | 3.99 | 2.00 | 0.0113870 |
| NUDT7 | 3.58 | 1.84 | 0.0113960 |
| MXRA7 | 3.00 | 1.59 | 0.0113980 |
| PLEKHG3 | 3.74 | 1.90 | 0.0114270 |
| ADAMTSL4 | 3.33 | 1.74 | 0.0114410 |
| HPGDS | 4.14 | 2.05 | 0.0114960 |
| RAP1GAP | -2.48 | -1.31 | 0.0115040 |
| PIK3R5 | 2.32 | 1.22 | 0.0115210 |
| COL13A1 | 4.02 | 2.01 | 0.0115450 |
| RARRES3 | 2.98 | 1.57 | 0.0115920 |
| GUCY1A3 | 3.79 | 1.92 | 0.0116180 |
| MASP2 | -3.71 | -1.89 | 0.0116620 |
| CD27 | 2.56 | 1.36 | 0.0116840 |
| LGALS1 | 2.39 | 1.26 | 0.0116870 |
| TNFRSF18 | 3.42 | 1.77 | 0.0117850 |
| SKA3 | 3.67 | 1.87 | 0.0119290 |
| PACSIN1 | 3.12 | 1.64 | 0.0119360 |
| ARRB1 | 3.44 | 1.78 | 0.0119950 |
| DISP2 | 2.78 | 1.47 | 0.0120220 |
| ANKRD35 | 4.07 | 2.02 | 0.0120490 |
| IRAK3 | 3.65 | 1.87 | 0.0121500 |
| LOC100506100 | 2.49 | 1.31 | 0.0123550 |
| LYPD2 | 4.06 | 2.02 | 0.0124960 |
| PIGK | 2.60 | 1.38 | 0.0125040 |
| ROPN1L | 3.65 | 1.87 | 0.0125280 |
| C20orf27 | 2.45 | 1.29 | 0.0125650 |
| MRAS | 3.86 | 1.95 | 0.0126030 |
| ETV4 | 4.04 | 2.01 | 0.0126760 |
| LOC100506844 | 3.05 | 1.61 | 0.0127420 |
| BTN3A2 | 2.43 | 1.28 | 0.0130590 |
| DHRS4-AS1 | 2.73 | 1.45 | 0.0130620 |
| TARP | 3.83 | 1.94 | 0.0130700 |
| FAM186B | -3.53 | -1.82 | 0.0131450 |
| RAG1 | 3.99 | 1.99 | 0.0131580 |
| HDC | 3.28 | 1.71 | 0.0131650 |
| PCGF6 | 2.38 | 1.25 | 0.0131920 |
| SLC27A4 | 2.36 | 1.24 | 0.0132300 |
| CASP5 | 3.43 | 1.78 | 0.0133310 |
| ND6 | -2.51 | -1.33 | 0.0133330 |
| CDC25C | 3.64 | 1.87 | 0.0133520 |
| LOC100507642 | 3.45 | 1.79 | 0.0133580 |
| IGSF10 | 3.94 | 1.98 | 0.0133700 |
| FDXR | 2.39 | 1.26 | 0.0134440 |
| GALK1 | 2.30 | 1.20 | 0.0135360 |
| PRDX2 | 3.46 | 1.79 | 0.0136100 |
| GALNT6 | 2.30 | 1.20 | 0.0137410 |
| TUBG1 | 2.52 | 1.33 | 0.0137530 |
| NLRP7 | 3.99 | 2.00 | 0.0138360 |
| PANK1 | 2.57 | 1.36 | 0.0138480 |
| CYB561 | 3.18 | 1.67 | 0.0139010 |
| ND5 | -2.40 | -1.26 | 0.0139140 |
| IL18R1 | 3.02 | 1.59 | 0.0139510 |
| ARPC5 | 2.58 | 1.36 | 0.0139970 |
| TLR5 | 3.96 | 1.99 | 0.0140060 |
| DLX4 | 3.16 | 1.66 | 0.0140880 |
| KREMEN1 | 3.94 | 1.98 | 0.0141940 |
| CABP7 | -3.05 | -1.61 | 0.0141990 |
| TPSB2 | 3.96 | 1.99 | 0.0142620 |
| FHL3 | 2.96 | 1.57 | 0.0142690 |
| DOC2GP | 2.83 | 1.50 | 0.0144640 |
| APOBEC3C | 2.65 | 1.41 | 0.0145320 |
| SNORA80B | -2.47 | -1.31 | 0.0145680 |
| PLOD1 | 2.41 | 1.27 | 0.0145800 |
| FYB | 3.39 | 1.76 | 0.0145880 |
| CEBPD | 3.42 | 1.77 | 0.0146010 |
| CD38 | 3.49 | 1.80 | 0.0146540 |
| CCNJL | 3.45 | 1.79 | 0.0147100 |
| SFTPD | 3.88 | 1.96 | 0.0147500 |
| ASRGL1 | 3.52 | 1.82 | 0.0147760 |
| FGD6 | -2.32 | -1.21 | 0.0149270 |
| INE1 | -2.43 | -1.28 | 0.0149280 |
| CDK4 | 2.45 | 1.29 | 0.0149790 |
| HILPDA | 2.70 | 1.43 | 0.0150250 |
| RLN3 | -3.49 | -1.81 | 0.0150310 |
| SULF2 | 2.88 | 1.53 | 0.0150360 |
| ALKBH4 | 2.40 | 1.26 | 0.0150390 |
| TTK | 3.08 | 1.62 | 0.0150510 |
| TBX15 | -2.99 | -1.58 | 0.0150810 |
| SPA17 | 2.90 | 1.53 | 0.0151100 |
| CMPK2 | 3.77 | 1.92 | 0.0151300 |
| ADAMTS10 | -2.77 | -1.47 | 0.0151300 |
| LRRC20 | 3.04 | 1.61 | 0.0152070 |
| SCT | 3.62 | 1.86 | 0.0152380 |
| ST3GAL4 | 2.97 | 1.57 | 0.0152480 |
| NPTXR | 3.56 | 1.83 | 0.0152930 |
| TSEN54 | 2.30 | 1.20 | 0.0152930 |
| HOXB3 | 3.27 | 1.71 | 0.0154090 |
| LOC100289361 | 2.85 | 1.51 | 0.0154230 |
| NWD1 | -3.04 | -1.60 | 0.0154290 |
| MTMR11 | 2.67 | 1.42 | 0.0154990 |
| DNAJB8-AS1 | -3.49 | -1.80 | 0.0155550 |
| HIC2 | -2.39 | -1.26 | 0.0155690 |
| LOC100507540 | 2.88 | 1.53 | 0.0157880 |
| PCYOX1 | 2.67 | 1.42 | 0.0159020 |
| SPEG | 3.87 | 1.95 | 0.0159070 |
| ZSWIM4 | -2.45 | -1.29 | 0.0159770 |
| BTBD3 | 3.56 | 1.83 | 0.0160780 |
| IFI6 | 2.64 | 1.40 | 0.0161080 |
| CERCAM | 2.96 | 1.57 | 0.0162130 |
| PCNA | 2.38 | 1.25 | 0.0163020 |
| LIF | 3.39 | 1.76 | 0.0163280 |
| TJP3 | 3.47 | 1.80 | 0.0164260 |
| ZNF331 | -2.83 | -1.50 | 0.0164610 |
| C2orf48 | 3.72 | 1.90 | 0.0164920 |
| LINC00092 | 3.83 | 1.94 | 0.0165450 |
| CD36 | 3.41 | 1.77 | 0.0165850 |
| HFE | 3.55 | 1.83 | 0.0166110 |
| SNAI3 | 2.83 | 1.50 | 0.0166160 |
| SLC6A9 | 3.57 | 1.84 | 0.0167420 |
| ZBED8 | 2.93 | 1.55 | 0.0167880 |
| RAB3C | 3.79 | 1.92 | 0.0168690 |
| ITPR1-AS1 | 3.68 | 1.88 | 0.0169370 |
| MGST1 | 3.52 | 1.81 | 0.0169970 |
| POC1A | 2.40 | 1.26 | 0.0170810 |
| SNORA15 | -2.84 | -1.51 | 0.0171450 |
| TUBB4A | 2.80 | 1.49 | 0.0171910 |
| FAM171A2 | 3.09 | 1.63 | 0.0172230 |
| LOC100506551 | -3.51 | -1.81 | 0.0172860 |
| TNFRSF10C | 3.80 | 1.93 | 0.0173630 |
| SULT1B1 | 3.80 | 1.93 | 0.0174490 |
| TMEM182 | 2.43 | 1.28 | 0.0174650 |
| KIF17 | -3.42 | -1.77 | 0.0175190 |
| TMOD1 | 3.45 | 1.78 | 0.0175490 |
| LOC554206 | 2.67 | 1.42 | 0.0175560 |
| CEP41 | 2.40 | 1.26 | 0.0176030 |
| MRVI1 | 2.99 | 1.58 | 0.0176700 |
| CD8A | 3.52 | 1.81 | 0.0176730 |
| FSIP1 | 3.04 | 1.60 | 0.0176750 |
| KLRF1 | 3.29 | 1.72 | 0.0177430 |
| ANKRD9 | 2.91 | 1.54 | 0.0177430 |
| ESCO2 | 3.42 | 1.77 | 0.0179660 |
| TMEM218 | 2.40 | 1.26 | 0.0181090 |
| CDK6 | 3.45 | 1.79 | 0.0182650 |
| OAS1 | 2.64 | 1.40 | 0.0183010 |
| ABCG2 | 3.14 | 1.65 | 0.0184990 |
| ISG15 | 2.64 | 1.40 | 0.0185360 |
| RILP | 2.61 | 1.38 | 0.0185400 |
| TLL2 | 3.12 | 1.64 | 0.0185500 |
| TNFSF13 | 2.60 | 1.38 | 0.0186800 |
| FLJ46906 | 3.46 | 1.79 | 0.0186830 |
| GPRC5C | 3.47 | 1.79 | 0.0187080 |
| PLA2G2D | 3.34 | 1.74 | 0.0187700 |
| MSX2P1 | 3.70 | 1.89 | 0.0188280 |
| NCR1 | 3.11 | 1.64 | 0.0190230 |
| EXO1 | 2.42 | 1.28 | 0.0190470 |
| LDB2 | 3.60 | 1.85 | 0.0190500 |
| PTPRQ | -3.56 | -1.83 | 0.0190540 |
| RRAS | 2.53 | 1.34 | 0.0191130 |
| PYCR1 | 2.88 | 1.52 | 0.0191240 |
| SPHK1 | 3.48 | 1.80 | 0.0193430 |
| LOC652276 | -2.68 | -1.42 | 0.0194380 |
| NLGN3 | -2.61 | -1.38 | 0.0194500 |
| PDK4 | 3.00 | 1.58 | 0.0194960 |
| UBASH3A | 3.41 | 1.77 | 0.0195060 |
| MAP3K12 | 2.76 | 1.47 | 0.0195640 |
| MPP1 | 3.02 | 1.59 | 0.0195920 |
| NEDD4 | 3.35 | 1.74 | 0.0196130 |
| EPHX1 | 2.68 | 1.42 | 0.0196600 |
| APOBEC3G | 2.35 | 1.23 | 0.0198930 |
| SPNS3 | 3.24 | 1.70 | 0.0202060 |
| SLC31A1 | 2.34 | 1.22 | 0.0203560 |
| ASIP | 2.45 | 1.30 | 0.0203700 |
| DYSF | 3.42 | 1.77 | 0.0204040 |
| LGMN | 2.65 | 1.41 | 0.0204070 |
| AK7 | 3.62 | 1.86 | 0.0204120 |
| GIMAP1 | 2.54 | 1.34 | 0.0204320 |
| TMTC1 | 3.68 | 1.88 | 0.0204370 |
| ERI2 | 2.41 | 1.27 | 0.0204590 |
| MYBL1 | 3.00 | 1.59 | 0.0205150 |
| PCDHGA8 | 3.62 | 1.86 | 0.0205290 |
| RYR1 | 2.35 | 1.23 | 0.0205460 |
| CKMT2-AS1 | 2.47 | 1.31 | 0.0205700 |
| PEX11G | 3.20 | 1.68 | 0.0205710 |
| RTN1 | 3.34 | 1.74 | 0.0205720 |
| RARA | -2.40 | -1.26 | 0.0206520 |
| MATN1 | -3.06 | -1.61 | 0.0206600 |
| NUAK1 | 3.50 | 1.81 | 0.0206690 |
| MGAT3 | 3.17 | 1.66 | 0.0207750 |
| ULK4P3 | 3.48 | 1.80 | 0.0207760 |
| TPPP | 3.39 | 1.76 | 0.0208570 |
| APOL1 | 2.36 | 1.24 | 0.0209200 |
| PTPN13 | 3.64 | 1.86 | 0.0209630 |
| PTPRU | -3.23 | -1.69 | 0.0210480 |
| JAKMIP1 | 3.48 | 1.80 | 0.0210660 |
| PRR5 | 2.32 | 1.21 | 0.0210910 |
| MEIS1 | 3.18 | 1.67 | 0.0211240 |
| HDDC3 | 2.36 | 1.24 | 0.0212460 |
| CPB2-AS1 | 2.84 | 1.50 | 0.0212480 |
| CD1C | 3.25 | 1.70 | 0.0212700 |
| ZFR2 | 3.57 | 1.84 | 0.0213120 |
| SIGMAR1 | 2.41 | 1.27 | 0.0213890 |
| PPP2R2B | 3.28 | 1.71 | 0.0214100 |
| ASB16 | -3.52 | -1.82 | 0.0214440 |
| WDFY3 | 3.40 | 1.76 | 0.0215760 |
| CYP1B1 | 3.55 | 1.83 | 0.0217080 |
| LDLR | 2.80 | 1.48 | 0.0217270 |
| MIDN | -2.41 | -1.27 | 0.0217500 |
| GUCY1B3 | 3.58 | 1.84 | 0.0218400 |
| MFGE8 | 3.33 | 1.74 | 0.0218630 |
| OLIG1 | 3.59 | 1.85 | 0.0220780 |
| ZNF432 | -2.33 | -1.22 | 0.0220780 |
| FGF8 | -3.61 | -1.85 | 0.0222380 |
| C1QTNF9B-AS1 | 2.38 | 1.25 | 0.0222840 |
| DPCD | 2.93 | 1.55 | 0.0223420 |
| IL1RN | 3.55 | 1.83 | 0.0223460 |
| SMAGP | 2.87 | 1.52 | 0.0224060 |
| P3H4 | 3.06 | 1.61 | 0.0224130 |
| SEMA3C | 3.41 | 1.77 | 0.0224910 |
| ZNF467 | 2.91 | 1.54 | 0.0226990 |
| SLC35G1 | 3.26 | 1.71 | 0.0227620 |
| TSPAN9 | 3.01 | 1.59 | 0.0229770 |
| CD300C | 3.20 | 1.68 | 0.0230110 |
| NDUFA8 | 2.50 | 1.32 | 0.0230160 |
| C5orf60 | -3.38 | -1.76 | 0.0230270 |
| CEACAM3 | 3.54 | 1.82 | 0.0230430 |
| MTL5 | 3.02 | 1.60 | 0.0230620 |
| KNDC1 | 3.47 | 1.80 | 0.0230720 |
| MIR4461 | -3.44 | -1.78 | 0.0232020 |
| CDA | 3.58 | 1.84 | 0.0232180 |
| LHX3 | -3.41 | -1.77 | 0.0232720 |
| RNF165 | 3.21 | 1.68 | 0.0232850 |
| CCDC74A | 3.27 | 1.71 | 0.0233350 |
| DCANP1 | 3.55 | 1.83 | 0.0233950 |
| RBAKDN | -3.32 | -1.73 | 0.0234780 |
| FLJ42627 | -3.09 | -1.63 | 0.0235090 |
| SPIB | 2.33 | 1.22 | 0.0235400 |
| SCML4 | 2.32 | 1.22 | 0.0235580 |
| DUBR | 3.20 | 1.68 | 0.0235830 |
| B3GNT8 | 3.51 | 1.81 | 0.0235990 |
| HOXC5 | 3.46 | 1.79 | 0.0235990 |
| FAM111B | -2.66 | -1.41 | 0.0236800 |
| MREG | 2.67 | 1.42 | 0.0237730 |
| TMEM163 | 3.38 | 1.76 | 0.0237940 |
| ST3GAL6 | 3.36 | 1.75 | 0.0238170 |
| EFNB1 | 2.56 | 1.35 | 0.0238170 |
| MESP1 | 2.98 | 1.57 | 0.0238740 |
| SEMA4C | 3.09 | 1.63 | 0.0239740 |
| S100A9 | 3.30 | 1.72 | 0.0242900 |
| CFAP58-AS1 | 3.54 | 1.82 | 0.0243620 |
| CFH | 3.50 | 1.81 | 0.0244490 |
| GGH | 2.85 | 1.51 | 0.0245200 |
| C10orf10 | -3.51 | -1.81 | 0.0245930 |
| KIAA1671 | 3.13 | 1.65 | 0.0246020 |
| GBP1 | 2.64 | 1.40 | 0.0246280 |
| APOBEC3A | 3.49 | 1.80 | 0.0247230 |
| OAF | 2.97 | 1.57 | 0.0248580 |
| SIPA1L2 | 3.10 | 1.63 | 0.0248790 |
| LOC101928823 | -3.45 | -1.79 | 0.0249420 |
| MAFB | 3.02 | 1.59 | 0.0249840 |
| BCDIN3D-AS1 | 2.82 | 1.50 | 0.0249950 |
| IFNG-AS1 | -3.46 | -1.79 | 0.0251360 |
| NCAM1 | 3.25 | 1.70 | 0.0252860 |
| OGFR-AS1 | -3.47 | -1.80 | 0.0254410 |
| CCDC177 | -3.50 | -1.81 | 0.0255950 |
| C19orf81 | 3.44 | 1.78 | 0.0256740 |
| SOWAHD | 2.32 | 1.22 | 0.0258510 |
| NMUR1 | 3.10 | 1.63 | 0.0258970 |
| HPGD | 3.43 | 1.78 | 0.0259140 |
| ABCB9 | 2.62 | 1.39 | 0.0259720 |
| MICAL2 | 3.10 | 1.63 | 0.0259960 |
| NREP | 3.15 | 1.65 | 0.0260110 |
| PTPRM | 3.22 | 1.69 | 0.0261040 |
| NEUROG2 | 3.49 | 1.80 | 0.0261140 |
| LOC113230 | 2.58 | 1.37 | 0.0263190 |
| CDC25A | 2.89 | 1.53 | 0.0264750 |
| TALDO1 | 2.53 | 1.34 | 0.0265000 |
| PDZD4 | 3.09 | 1.63 | 0.0265320 |
| AAMDC | 2.57 | 1.36 | 0.0265660 |
| HOMER3 | 2.33 | 1.22 | 0.0265920 |
| LINC00640 | -2.79 | -1.48 | 0.0266930 |
| MMP25 | 2.79 | 1.48 | 0.0267730 |
| GOLM1 | 2.88 | 1.52 | 0.0268340 |
| LYAR | 2.30 | 1.20 | 0.0268720 |
| BIVM | 2.35 | 1.23 | 0.0269340 |
| TMC4 | 2.97 | 1.57 | 0.0272390 |
| ITLN1 | 3.45 | 1.79 | 0.0273420 |
| C21orf62-AS1 | 2.36 | 1.24 | 0.0273700 |
| FAM47E | 3.44 | 1.78 | 0.0273970 |
| DPYSL4 | -3.28 | -1.71 | 0.0275910 |
| ZBTB7C | 3.43 | 1.78 | 0.0276490 |
| MOCOS | 2.92 | 1.54 | 0.0276540 |
| LOC100506299 | 3.45 | 1.78 | 0.0276830 |
| S100Z | 3.38 | 1.76 | 0.0279120 |
| SLC11A1 | 3.08 | 1.62 | 0.0279310 |
| NCKAP1 | 3.27 | 1.71 | 0.0280710 |
| TK1 | 2.80 | 1.48 | 0.0280930 |
| RMI2 | 2.40 | 1.26 | 0.0281140 |
| PLEKHD1 | -3.02 | -1.60 | 0.0281150 |
| FSCN1 | 2.70 | 1.44 | 0.0281170 |
| IL18RAP | 2.91 | 1.54 | 0.0281450 |
| LOC101927811 | 3.43 | 1.78 | 0.0281640 |
| PKP4 | 3.40 | 1.77 | 0.0282010 |
| SLA2 | 2.64 | 1.40 | 0.0282850 |
| LILRA2 | 3.23 | 1.69 | 0.0283740 |
| PTAFR | 2.58 | 1.37 | 0.0284780 |
| ALDH5A1 | 2.34 | 1.23 | 0.0284890 |
| SIRPB2 | 3.42 | 1.77 | 0.0285340 |
| ITLN2 | -3.37 | -1.75 | 0.0285630 |
| ATP8B4 | 3.07 | 1.62 | 0.0286170 |
| APBA2 | 2.82 | 1.49 | 0.0286560 |
| HRASLS2 | 3.15 | 1.66 | 0.0287390 |
| SERP2 | 3.40 | 1.77 | 0.0287860 |
| GPNMB | 3.27 | 1.71 | 0.0289360 |
| EPHB1 | 3.30 | 1.72 | 0.0289690 |
| ITM2C | 2.59 | 1.37 | 0.0289800 |
| MMP15 | 2.66 | 1.41 | 0.0290310 |
| FADS2 | 3.02 | 1.60 | 0.0291300 |
| SLC4A4 | 3.16 | 1.66 | 0.0291730 |
| DQX1 | 3.31 | 1.73 | 0.0291940 |
| TPSD1 | 3.39 | 1.76 | 0.0292700 |
| TRIM47 | 2.61 | 1.39 | 0.0293720 |
| GADD45G | 3.31 | 1.73 | 0.0294110 |
| BAALC | 3.23 | 1.69 | 0.0296010 |
| PLB1 | 2.96 | 1.56 | 0.0296370 |
| LOC101927045 | 2.46 | 1.30 | 0.0297270 |
| DRAXIN | 3.25 | 1.70 | 0.0297630 |
| LOC101929241 | 3.36 | 1.75 | 0.0298750 |
| PARS2 | 2.80 | 1.49 | 0.0298890 |
| RNASEH2A | 2.33 | 1.22 | 0.0298930 |
| SLC8A1 | 3.07 | 1.62 | 0.0299230 |
| CCDC102A | 2.91 | 1.54 | 0.0302080 |
| CAPN11 | 3.37 | 1.75 | 0.0303810 |
| RET | 3.37 | 1.75 | 0.0303990 |
| GPR171 | 3.17 | 1.67 | 0.0305010 |
| AMY2A | -3.13 | -1.64 | 0.0305550 |
| VENTX | 3.26 | 1.71 | 0.0305650 |
| C9orf64 | 2.52 | 1.33 | 0.0305870 |
| IL13RA1 | 2.61 | 1.39 | 0.0306240 |
| EIF4E3 | 2.86 | 1.52 | 0.0307730 |
| KLRC2 | 3.26 | 1.70 | 0.0308710 |
| GSTM3 | 3.28 | 1.71 | 0.0309010 |
| ACPP | 3.01 | 1.59 | 0.0310240 |
| TNNT1 | 3.27 | 1.71 | 0.0310550 |
| LOC100289511 | -2.68 | -1.42 | 0.0310600 |
| MIXL1 | 3.17 | 1.66 | 0.0311270 |
| AP1S1 | 2.30 | 1.20 | 0.0311310 |
| PRKCQ | 3.05 | 1.61 | 0.0311540 |
| CDCP1 | 3.26 | 1.71 | 0.0311680 |
| GINS1 | 2.32 | 1.22 | 0.0312960 |
| NSG1 | 3.30 | 1.72 | 0.0313780 |
| TMEM204 | 3.04 | 1.60 | 0.0314550 |
| LOC158960 | 2.70 | 1.43 | 0.0316200 |
| SDR9C7 | -3.29 | -1.72 | 0.0317990 |
| RAB9B | 2.57 | 1.36 | 0.0319340 |
| LST1 | 2.85 | 1.51 | 0.0322100 |
| ZNF404 | 2.69 | 1.43 | 0.0322520 |
| TPX2 | 2.35 | 1.23 | 0.0322800 |
| CAMP | 3.20 | 1.68 | 0.0324000 |
| PTMS | 2.31 | 1.21 | 0.0325330 |
| CD101 | 2.87 | 1.52 | 0.0326470 |
| CYSLTR2 | 3.16 | 1.66 | 0.0327810 |
| PLTP | 2.91 | 1.54 | 0.0328630 |
| FOXN3-AS1 | 2.34 | 1.23 | 0.0330420 |
| DOK5 | 2.82 | 1.50 | 0.0330590 |
| E2F2 | 2.82 | 1.50 | 0.0331750 |
| C11orf74 | 3.22 | 1.69 | 0.0332510 |
| SNORD8 | -2.91 | -1.54 | 0.0336120 |
| DSG2 | 3.29 | 1.72 | 0.0337620 |
| PTPRN2 | 2.74 | 1.45 | 0.0338950 |
| PLD4 | 2.92 | 1.54 | 0.0338970 |
| RAPH1 | 3.11 | 1.64 | 0.0339870 |
| ICAM4 | 3.22 | 1.69 | 0.0339980 |
| CEBPE | 3.16 | 1.66 | 0.0340240 |
| LOC101929567 | 3.29 | 1.72 | 0.0341050 |
| UBE2T | 2.60 | 1.38 | 0.0341190 |
| MIR6819 | -2.57 | -1.36 | 0.0341570 |
| CXCL2 | -2.94 | -1.56 | 0.0342140 |
| HPDL | 2.41 | 1.27 | 0.0342390 |
| KIR2DS4 | 3.27 | 1.71 | 0.0344730 |
| B9D1 | 2.54 | 1.35 | 0.0345570 |
| KIF18B | 2.84 | 1.50 | 0.0345940 |
| S100A2 | 2.77 | 1.47 | 0.0346680 |
| MINPP1 | 2.36 | 1.24 | 0.0350200 |
| TMPRSS4 | -3.20 | -1.68 | 0.0355390 |
| LOC100129083 | 2.99 | 1.58 | 0.0358680 |
| SHISA4 | 2.81 | 1.49 | 0.0358740 |
| COA3 | 2.31 | 1.21 | 0.0359280 |
| SGCA | 2.35 | 1.23 | 0.0359880 |
| THBS4 | -2.89 | -1.53 | 0.0359930 |
| LACTB2 | 2.55 | 1.35 | 0.0360670 |
| CLEC10A | 3.10 | 1.63 | 0.0360840 |
| RALGAPA1P | -3.13 | -1.65 | 0.0361010 |
| RSAD2 | 3.11 | 1.64 | 0.0362230 |
| PPP1R36 | 3.09 | 1.63 | 0.0362940 |
| FOXRED2 | 2.31 | 1.21 | 0.0362960 |
| FERMT2 | -2.88 | -1.53 | 0.0362960 |
| TMEM173 | 2.46 | 1.30 | 0.0363190 |
| FAM101B | 2.77 | 1.47 | 0.0365870 |
| PRKCH | 2.92 | 1.55 | 0.0369320 |
| CPNE6 | 3.22 | 1.69 | 0.0369820 |
| ENPP6 | 3.23 | 1.69 | 0.0371410 |
| SPNS2 | 2.30 | 1.20 | 0.0371840 |
| CPA3 | 3.11 | 1.64 | 0.0373300 |
| GPR150 | 3.22 | 1.69 | 0.0373560 |
| OSCAR | 2.79 | 1.48 | 0.0374320 |
| CMYA5 | -2.32 | -1.22 | 0.0375750 |
| RWDD2B | 2.45 | 1.29 | 0.0377530 |
| LOC102724316 | 2.97 | 1.57 | 0.0377610 |
| PSORS1C3 | -3.10 | -1.63 | 0.0377610 |
| SIRPG | 3.17 | 1.66 | 0.0378080 |
| NHLRC1 | 3.04 | 1.60 | 0.0378750 |
| SMCO4 | 3.01 | 1.59 | 0.0379630 |
| ZAK | 2.89 | 1.53 | 0.0380870 |
| MAF | 2.96 | 1.56 | 0.0380920 |
| LEPR | 2.55 | 1.35 | 0.0382450 |
| KIAA1614 | -3.15 | -1.66 | 0.0383150 |
| CEP19 | 2.61 | 1.39 | 0.0385000 |
| JAKMIP2 | 3.11 | 1.64 | 0.0385050 |
| TSPAN12 | -3.17 | -1.67 | 0.0388100 |
| TLE6 | 2.80 | 1.49 | 0.0388770 |
| EVA1C | 2.97 | 1.57 | 0.0390100 |
| TMEM144 | 3.12 | 1.64 | 0.0390960 |
| TNFAIP2 | 2.58 | 1.37 | 0.0391650 |
| MT1E | 3.02 | 1.59 | 0.0391670 |
| ADGRV1 | -3.14 | -1.65 | 0.0392300 |
| COL2A1 | -2.98 | -1.57 | 0.0393870 |
| NEURL1 | 2.88 | 1.53 | 0.0394070 |
| NR6A1 | 2.53 | 1.34 | 0.0394240 |
| MGAM | 2.64 | 1.40 | 0.0395490 |
| CTXN1 | 3.15 | 1.65 | 0.0396160 |
| PHKG1 | -2.38 | -1.25 | 0.0396390 |
| SOCS2-AS1 | 2.64 | 1.40 | 0.0396920 |
| FNIP2 | 2.86 | 1.52 | 0.0397330 |
| RUNX2 | 2.90 | 1.53 | 0.0398230 |
| RBFOX2 | 3.14 | 1.65 | 0.0399920 |
| NCEH1 | 2.36 | 1.24 | 0.0400430 |
| UGDH | 2.43 | 1.28 | 0.0400670 |
| CHI3L2 | 2.77 | 1.47 | 0.0401270 |
| ZNF697 | 3.05 | 1.61 | 0.0402720 |
| FCGR1A | 3.04 | 1.60 | 0.0403910 |
| CYP27B1 | -2.36 | -1.24 | 0.0405600 |
| CALCRL | 3.15 | 1.66 | 0.0406520 |
| PDCD1LG2 | 3.05 | 1.61 | 0.0406670 |
| HN1 | 2.38 | 1.25 | 0.0406770 |
| TRPC2 | 3.15 | 1.65 | 0.0408230 |
| SLC16A14 | 2.78 | 1.47 | 0.0411310 |
| MIR6878 | -2.82 | -1.50 | 0.0411660 |
| STAC | 3.06 | 1.62 | 0.0411700 |
| SMO | -2.65 | -1.40 | 0.0412510 |
| LCK | 2.47 | 1.30 | 0.0412930 |
| PLAU | 2.70 | 1.43 | 0.0415120 |
| ELK2AP | -2.57 | -1.36 | 0.0415660 |
| SCML2 | 2.95 | 1.56 | 0.0416920 |
| KIF20A | 2.89 | 1.53 | 0.0417090 |
| LOC102724927 | 3.14 | 1.65 | 0.0417440 |
| EOMES | 2.87 | 1.52 | 0.0417460 |
| LOC100996455 | 3.11 | 1.64 | 0.0417510 |
| UQCRBP1 | -2.59 | -1.37 | 0.0417690 |
| PRKCQ-AS1 | 2.97 | 1.57 | 0.0418870 |
| EHHADH | 2.62 | 1.39 | 0.0419020 |
| FCGR3B | 3.08 | 1.62 | 0.0422270 |
| IFI27 | 2.77 | 1.47 | 0.0424200 |
| CACNA1A | 2.51 | 1.33 | 0.0424440 |
| CDCA2 | 2.82 | 1.50 | 0.0425140 |
| SPDYA | -2.96 | -1.56 | 0.0426530 |
| CCL25 | 3.08 | 1.62 | 0.0427280 |
| GPR156 | -2.89 | -1.53 | 0.0428170 |
| CEP170P1 | -2.81 | -1.49 | 0.0429160 |
| ERRFI1 | 2.73 | 1.45 | 0.0430380 |
| EDARADD | 2.73 | 1.45 | 0.0431190 |
| DKKL1 | 2.50 | 1.32 | 0.0433140 |
| LBX2 | 2.79 | 1.48 | 0.0433470 |
| DNAJC27-AS1 | 2.79 | 1.48 | 0.0433530 |
| RAPGEF2 | -2.37 | -1.24 | 0.0433620 |
| TLR3 | 3.09 | 1.63 | 0.0434860 |
| GCAT | 2.68 | 1.42 | 0.0436510 |
| DPP4 | 2.42 | 1.28 | 0.0437870 |
| CLEC1A | 3.08 | 1.63 | 0.0439210 |
| LRP11 | 2.84 | 1.51 | 0.0439250 |
| GNGT2 | 2.94 | 1.56 | 0.0441530 |
| CD3E | 2.68 | 1.42 | 0.0443470 |
| LINC00239 | 3.08 | 1.62 | 0.0444990 |
| LRFN3 | 3.07 | 1.62 | 0.0445540 |
| LHX4 | -2.89 | -1.53 | 0.0447800 |
| XCL2 | 2.66 | 1.41 | 0.0448170 |
| EPHA2 | 2.72 | 1.44 | 0.0449270 |
| EPHB3 | 3.05 | 1.61 | 0.0449680 |
| RGN | -2.66 | -1.41 | 0.0450030 |
| GAD2 | -2.97 | -1.57 | 0.0450430 |
| CNTNAP2 | -3.00 | -1.59 | 0.0451220 |
| TIGIT | 2.41 | 1.27 | 0.0451250 |
| MEST | 2.52 | 1.33 | 0.0451380 |
| ADAMTS1 | 2.86 | 1.51 | 0.0452820 |
| ROCK1P1 | -2.57 | -1.36 | 0.0452960 |
| MEFV | 2.75 | 1.46 | 0.0453180 |
| KIAA2022 | -2.98 | -1.57 | 0.0453470 |
| ANXA3 | 3.07 | 1.62 | 0.0455080 |
| AOC4P | -2.85 | -1.51 | 0.0455190 |
| SNORD12C | -2.67 | -1.42 | 0.0455660 |
| EPS8 | 2.90 | 1.54 | 0.0458250 |
| SMARCD3 | 2.63 | 1.40 | 0.0458610 |
| LOC102724163 | 2.51 | 1.33 | 0.0460820 |
| PRICKLE4 | 2.36 | 1.24 | 0.0461320 |
| SCLY | 2.77 | 1.47 | 0.0461380 |
| SFRP2 | -2.97 | -1.57 | 0.0462030 |
| RTN4R | 2.87 | 1.52 | 0.0462270 |
| IFIT3 | 2.89 | 1.53 | 0.0462700 |
| EPHA4 | 2.49 | 1.32 | 0.0463380 |
| CACNA1B | -3.00 | -1.58 | 0.0463390 |
| DUSP19 | 2.68 | 1.42 | 0.0463910 |
| CD3EAP | 2.34 | 1.23 | 0.0465000 |
| PNCK | -2.90 | -1.54 | 0.0467980 |
| HAVCR1 | -2.48 | -1.31 | 0.0468450 |
| SFTPB | 2.96 | 1.56 | 0.0469220 |
| LOC101928847 | 2.95 | 1.56 | 0.0469320 |
| BAIAP2-AS1 | 3.03 | 1.60 | 0.0469390 |
| ENG | 2.67 | 1.42 | 0.0473660 |
| MIR219A1 | -2.97 | -1.57 | 0.0474900 |
| IFNA5 | -3.04 | -1.60 | 0.0475850 |
| IGSF6 | 2.98 | 1.58 | 0.0477390 |
| TSKS | 3.02 | 1.59 | 0.0477620 |
| MYO6 | 2.80 | 1.49 | 0.0478140 |
| LINC01137 | 2.62 | 1.39 | 0.0478700 |
| SLCO4C1 | 2.84 | 1.51 | 0.0479200 |
| IL1RL1 | 2.93 | 1.55 | 0.0479390 |
| MIR210HG | 2.62 | 1.39 | 0.0479620 |
| FUT3 | -2.85 | -1.51 | 0.0480570 |
| ZAR1L | -2.75 | -1.46 | 0.0481020 |
| VSIG8 | -2.99 | -1.58 | 0.0481840 |
| LDB3 | -2.53 | -1.34 | 0.0482980 |
| LOC100996324 | 2.90 | 1.53 | 0.0483740 |
| LOXHD1 | 3.03 | 1.60 | 0.0485980 |
| TBR1 | -2.90 | -1.54 | 0.0488090 |
| CCDC34 | 2.40 | 1.27 | 0.0492070 |
| RAB40AL | 2.84 | 1.51 | 0.0492530 |
| PTGFRN | 2.94 | 1.56 | 0.0492560 |
| MIF | 2.49 | 1.32 | 0.0493510 |
| DCBLD2 | 3.02 | 1.59 | 0.0494040 |
| ASB13 | 2.75 | 1.46 | 0.0498390 |
