# Supplementary material for: High expression level of ROR1 and ROR1-signaling associates with venetoclax resistance in chronic lymphocytic leukemia
Source: Leukemia. 2022 Apr 13;36(6):1609–18. doi: 10.1038/s41375-022-01543-y (PMC9162914; doi:10.1038/s41375-022-01543-y)
Supplement: Supplementary file 3 — Table S3 [file 41375_2022_1543_MOESM3_ESM.docx]

| Gene Sets | SIZE | ES | NES | NOM p-val | FDR q-val |
| --- | --- | --- | --- | --- | --- |
| NF-κB JASPAR PREDICTED TRANSCRIPTION FACTORS TARGETS (37, 42) | 2962 | 0.07 | 4.15 | 0.000 | 0.000 |
| GO_NON_CANONICAL_WNT_SIGNALING_PATHWAY (38) | 130 | 0.24 | 3.20 | 0.000 | 0.000 |
| NF-κB TARGET GENES (40) | 58 | 0.32 | 2.85 | 0.000 | 0.000 |
| NF-κB TARGET GENES (41) | 84 | 0.26 | 2.73 | 0.000 | 0.000 |
| GO_CANONICAL_WNT_SIGNALING_PATHWAY (38) | 84 | 0.12 | 1.26 | 0.262 | 0.284 |

**Table S3.** GSEA on the transcriptomes of MEC1-ROR1 versus MEC1, evaluating for differences in the expression of NF-κB target genes and of genes induced by canonical or non-canonical Wnt signaling pathway.(37, 38, 40-42) Gene-set size (SIZE), enrichment score (ES), normalized ES (NES), nominal p value (NOM p-val), and FDR q value (FDR q) are indicated.
