# Supplementary material for: High expression level of ROR1 and ROR1-signaling associates with venetoclax resistance in chronic lymphocytic leukemia
Source: Leukemia. 2022 Apr 13;36(6):1609–18. doi: 10.1038/s41375-022-01543-y (PMC9162914; doi:10.1038/s41375-022-01543-y)
Supplement: Supplementary file 4 — Table S4 [file 41375_2022_1543_MOESM4_ESM.docx]

| Sample Code | *BCL2* variants detected at SC1 | *BCL2* variants detected  at SC2 |
| --- | --- | --- |
| CLL1 | not detected | *BCL2* G101V (11.9%) |
| CLL2 | not detected | *BCL2* G101V (3.8%) |
| CLL3 | not detected | *BCL2* A113P (49.3%) |
| CLL4 | not detected | not detected |
| CLL5 | not detected | *BCL2* G101V (16.1%), *BCL2* A113G (1.5%) |
| CLL6 | not detected | not detected |
| CLL7 | not detected | not detected |

**Table S4.** *BCL2* mutations observed in CLL cells of 7 patients (CLL1-7) with persistent MRD after >1 year on venetoclax-based therapy at sample collection (SC) prior to therapy (SC1) or at MRD progression on therapy (SC2).
