# Supplementary material for: High expression level of ROR1 and ROR1-signaling associates with venetoclax resistance in chronic lymphocytic leukemia
Source: Leukemia. 2022 Apr 13;36(6):1609–18. doi: 10.1038/s41375-022-01543-y (PMC9162914; doi:10.1038/s41375-022-01543-y)
Supplement: Supplementary file 5 — Supplemental Figure Legends [file 41375_2022_1543_MOESM5_ESM.pdf]

Figure S1

Absolute CLL count prior to venetoclax therapy (t1), after 1 year (t2) of venetoclax therapy, at MRD progression on venetoclax therapy (t3), and at two consecutive time points after MRD progression (t4-t5) of 7 patients on continued venetoclax therapy (CLL1-7). The first sample collection (SC1), prior to the start of venetoclax therapy (V-Rx), and after more than 1 year of venetoclax therapy (second sample collection 2 (SC2) are indicated above.

Figure S2

(A) GSEA on the transcriptomes of 24 CLL samples evaluating for ROR1^{Hi} versus ROR1^{Lo} differences in the expression of ROR1-regulated target genes. Gene-set size (SIZE), enrichment score, normalized ES (NES), nominal p value (NOM p-val), and FDR q value (FDR q) are indicated. **(B)** Volcano plot showing differences in gene expression between ROR1^{Hi} versus ROR1^{Lo}. The log2 of the fold change (log2 Fold Change) is on the X axis, and the negative log10 of p-value (-log10 p-Value) is on the Y axis. Vertical dashed lines indicate fold change of 1.2 and -1.2 respectively. Horizontal dashed line indicates a p-value of 0.05. Each dot represents a gene within the comparison performed. The coloring on the dots reflects whether each gene is significantly overexpressed (green) or underexpressed (purple) in ROR1^{Hi} versus ROR1^{Lo}, and those in black are genes that were not significantly overexpressed or under-expressed in ROR1^{Hi} versus ROR1^{Lo}. The significant overexpression of *BCL2L1* in ROR1^{Hi} is indicated.

Figure S3

BCL-XL protein expression levels assessed by immunoblot analysis on MEC1 or MEC1-ROR1 cells following transfection with BCL2^{WT} or each of the BCL2 variants, as indicated. The membranes were probed with a monoclonal antibody specific for BCL-XL or tubulin, as indicated on the left margin. The density of the β-actin band was used to normalize band density

for BCL-XL in each sample. The IOD ratios of the band densities of BCL-XL/β-actin for each sample are indicated at the bottom of BCL-XL immunoblots.
