# Supplementary material for: High expression level of ROR1 and ROR1-signaling associates with venetoclax resistance in chronic lymphocytic leukemia
Source: Leukemia. 2022 Apr 13;36(6):1609–18. doi: 10.1038/s41375-022-01543-y (PMC9162914; doi:10.1038/s41375-022-01543-y)
Supplement: Supplementary file 6 — Supplemental Figures [file 41375_2022_1543_MOESM6_ESM.pdf]

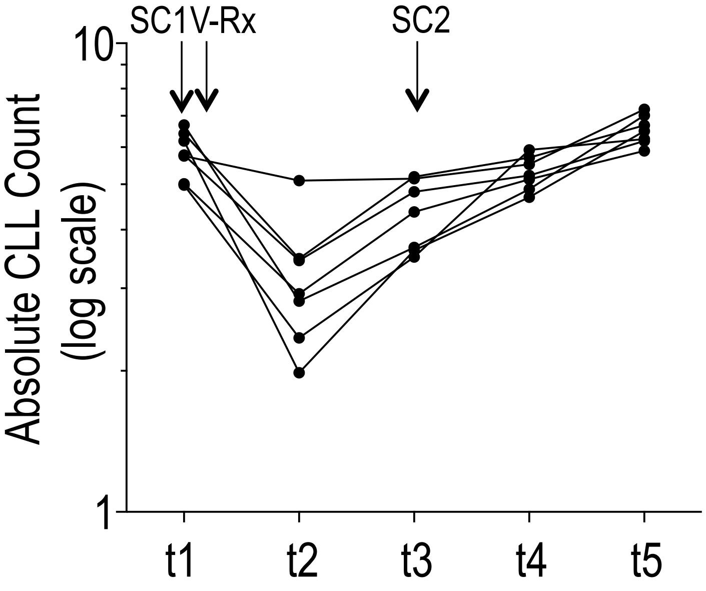

Figure S1

A

Gene Sets	SIZE	ES	NES	NOM p-val	FDR q-val
ERK1/2 TARGET GENES	6483	0.06	4.03	0.000	0.000
NF-κB HALLMARK	192	0.18	2.89	0.000	0.000
NF-κB TARGET GENES	53	0.13	1.13	0.165	0.145

B

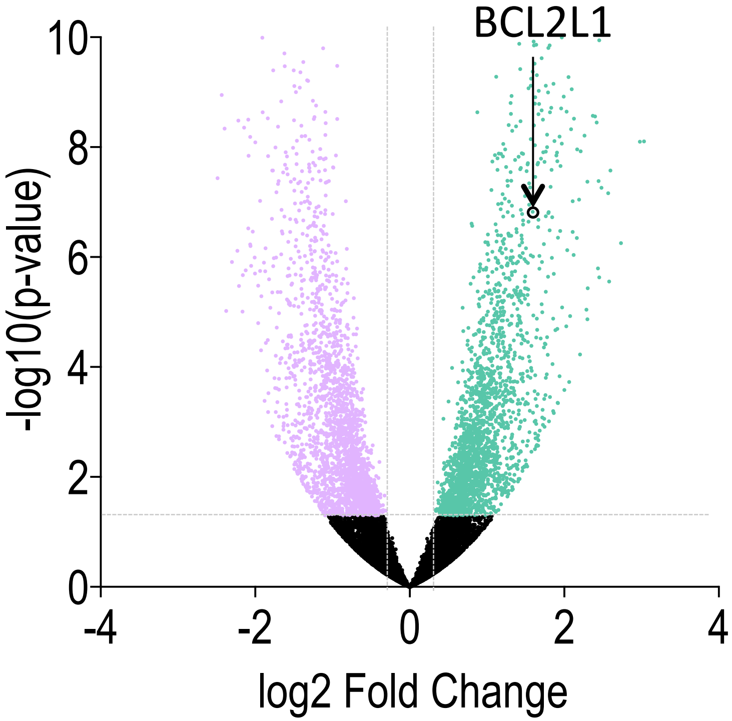

Figure S2

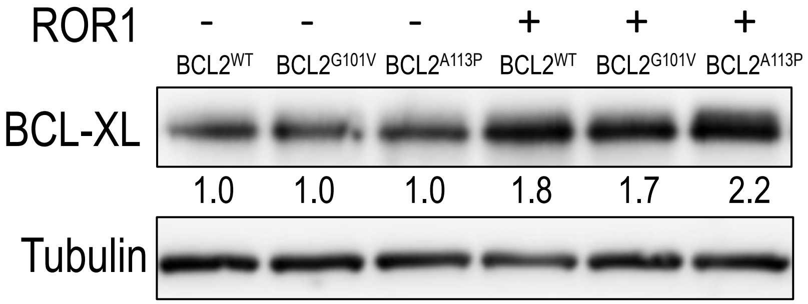

ROR1
-
-
-
+
+
+
BCL2WT
BCL2G101V
BCL2A113P
BCL2WT
BCL2G101V
BCL2A113P
BCL-XL
1.0
1.0
1.0
1.8
1.7
2.2
Tubulin

Figure S3
